# High‐Performance Ambipolar and n‐Type Emissive Semiconductors Based on Perfluorophenyl‐Substituted Perylene and Anthracene

**DOI:** 10.1002/advs.202300530

**Published:** 2023-03-26

**Authors:** Liangliang Chen, Zhengsheng Qin, Han Huang, Jing Zhang, Zheng Yin, Xiaobo Yu, Xi‐sha Zhang, Cheng Li, Guanxin Zhang, Miaofei Huang, Huanli Dong, Yuanping Yi, Lang Jiang, Hongbing Fu, Deqing Zhang

**Affiliations:** ^1^ Beijing National Laboratory for Molecular Sciences CAS Key Laboratory of Organic Solids Institute of Chemistry Chinese Academy of Sciences Beijing 100190 P. R. China; ^2^ School of Chemical Science University of Chinese Academy of Sciences Beijing 100049 P. R. China; ^3^ Beijing Key Laboratory for Optical Materials and Photonic Devices Department of Chemistry Capital Normal University Beijing 100048 P. R. China

**Keywords:** ambipolar semiconductors, emissive semiconductors, n‐type semiconductors, organic light‐emitting transistors

## Abstract

Emissive organic semiconductors are highly demanding for organic light‐emitting transistors (OLETs) and electrically pumped organic lasers (EPOLs). However, it remains a great challenge to obtain organic semiconductors with high carrier mobility and high photoluminescence quantum yield simultaneously. Here, a new design strategy is reported for highly emissive ambipolar and even n‐type semiconductors by introducing perfluorophenyl groups into polycyclic aromatic hydrocarbons such as perylene and anthracene. The results reveal that 3,9‐diperfluorophenyl perylene (**5FDPP**) exhibits the ambipolar semiconducting property with hole and electron mobilities up to 0.12 and 1.89 cm^2^ V^−1^ s^−1^, and a photoluminescence quantum yield of 55%. One of the crystal forms of **5FDPA** exhibits blue emission with an emission quantum yield of 52% and simultaneously shows the n‐type semiconducting property with an electron mobility up to 2.65 cm^2^ V^−1^ s^−1^, which is the highest value among the reported organic emissive n‐type semiconductors. Furthermore, crystals of **5FDPP** are utilized to fabricate OLETs by using Ag as source–drain electrodes. The electroluminescence is detected in the transporting channels with an external quantum efficiency (EQE) of up to 2.2%, and the current density is up to 145 kA cm^−2^, which are among the highest values for single‐component OLETs with symmetric electrodes.

## Graphical abstract

3,9‐diperfluorophenyl perylene (**5FDPP**) exhibits the ambipolar semiconducting property with hole and electron mobilities up to 0.12 and 1.89 cm^2^ V^−1^ s^−1^, and a photoluminescence quantum yield (PLQY) of 55%, while 2,6‐diperfluorophenyl anthracene (**5FDPA**) exhibits the n‐type semiconducting property with an electron mobility of up to 2.65 cm^2^ V^−1^ s^−1^ and a PLQY of 52%. **5FDPP**‐based organic light‐emitting transistor (OLET) devices display spatially controlled electroluminescence with an external quantum efficiency (EQE) of 2.2%.

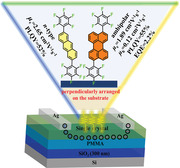

## 1 Introduction

Emissive organic semiconductors have received increasing attention because they have great potential applications in organic light‐emitting transistors (OLETs)^[^ ^1^ ^]^ and electrically pumped organic lasers (EPOLs).^[^ ^2^ ^]^ However, it remains challenging to retain high carrier mobility and high photoluminescence quantum yield (PLQY) simultaneously in the aggregate state.^[^ ^3^ ^]^ This is because high carrier mobility usually requires compact packing with strong intermolecular interactions of conjugated molecular systems,^[^ ^4^ ^]^ but these molecules usually show weak emissions in the aggregated states due to quenching of the excited states by intermolecular tightly packing^[^ ^5^ ^]^ or singlet fissions.^[^ ^6^ ^]^ Meanwhile, high PLQY usually demands weak intermolecular interaction and twisting structures,^[^ ^7^ ^]^ which are not beneficial for intermolecular dense packing and thus charge transporting.^[^ ^8^ ^]^ Consequently, this contradiction makes it hard to design highly emissive organic semiconductors with high charge mobilities. Nevertheless, several emissive p‐type organic semiconductors have been reported. For instance, Perepichka and co‐workers reported 2‐(4‐hexylphenylvinyl)anthracene as the emissive p‐type semiconductor with a high PLQY of up to 70% and a hole mobility of up to 2.6 cm^2^ V^−1^ s^−1^ in 2012.^[^ ^9^ ^]^ Hu and co‐workers found that 2,6‐diphenylanthracene shows blue emission with a PLQY of 41.2% and p‐type semiconducting property with a hole mobility of 34 cm^2^ V^−1^ s^−1^.^[^ ^10^ ^]^ More 2,6‐ and 9,10‐substituted anthracene compounds were found to be highly emissive p‐type semiconductors.^[^ ^11^ ^]^ Besides, pyrene derivatives were also reported to show emissive p‐type charge‐transporting behavior by Tian and co‐workers^[^ ^12^ ^]^ and some of us as well.^[^ ^13^ ^]^ Dong and co‐workers have recently reported 2,7‐diphenyl‐9*H*‐fluorene exhibiting high performances in charge transporting and organic lasing.^[^ ^14^ ^]^ In comparison, organic materials with high electron mobility and even ambipolar transporting and strong emission in the aggregate state are still limited.^[^ ^15^ ^]^ Perfluorohexylquaterthiophene was found to show n‐type semiconducting and emissive properties, but the electron mobility and emission quantum yield were low.^[^ ^16^ ^]^ Certain derivatives of naphthalene diimide and perylene diimide were reported by Tang and co‐workers^[^ ^17^ ^]^ and some of us^[^ ^18^ ^]^ to show dual functions with tunable emission colors, but they exhibited low electron mobilities. Ma and co‐workers reported the crystals of 1,4‐bis(2‐cyano‐2‐phenylethenyl)benzene exhibited balanced hole and electron mobilities and high photoluminescence.^[^ ^15^, ^19^ ^]^ But most of the reported emissive n‐type or ambipolar organic semiconductors suffer from either low mobility or low PLQY (Figure S20, Supporting Information).^[^ ^20^ ^]^ Meanwhile, the OLET devices, in which unsymmetric source–drain electrodes were used, with emissive semiconductors were fabricated and investigated.^[^ ^19^, ^21^ ^]^ However, the devices of unsymmetric source–drain electrodes require complicated fabricating procedures. Therefore, developing intrinsically ambipolar emissive semiconductors is essential for simplifying the structure of OLET devices and promoting practical applications.^[^ ^20c^ ^]^

**Scheme 1:**
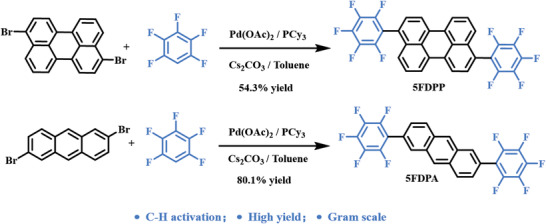
The synthetic routes of **5FDPP** and **5FDPA**.

It is known that the connection of perfluorophenyl groups to small‐molecule p‐type organic semiconductors can yield ambipolar or n‐type organic semiconductors.^[^ ^22^ ^]^ Considering perylene and anthracene as excellent emissive moieties,^[^ ^23^ ^]^ we incorporate perfluorophenyl groups into perylene and anthracene aiming to obtain ambipolar and n‐type emissive semiconductors. Both 3,9‐diperfluorophenyl perylene (**5FDPP**, **Scheme** **1**) and 2,6‐diperfluorophenyl anthracene (**5FDPA**, Scheme 1) were synthesized and investigated. The results reveal that **5FDPP** shows a typical ambipolar semiconducting property with hole and electron mobilities up to 0.12 and 1.89 cm^2^ V^−1^ s^−1^, respectively, and strong emission peaking at around 537 nm with a quantum yield of 55% in the solid state simultaneously. In comparison, **5FDPA** behaves as an emissive n‐type semiconductor with high PLQY and electron mobility. Interestingly, **5FDPA** exhibits polymorphism. The blue‐emissive crystals of **5FDPA** (referred to as crystals‐B) display a PLQY of 52% and behave as a n‐type semiconductor with an electron mobility of up to 2.65 cm^2^ V^−1^ s^−1^. Remarkably, crystals‐B can be transformed into green‐emissive crystals (referred to as crystals‐G) by cooling at 78 K, but crystals‐G show low electron mobility (0.17 cm^2^ V^−1^ s^−1^). In addition, blue‐emissive crystals of **5FDPA,** which were crystallized from CHCl_3_ (referred to as crystals‐C), were also obtained, but they exhibit poor semiconducting properties. Furthermore, the blue‐emissive crystalline samples (crystals‐B) of **5FDPA** can be converted into the green‐emissive samples by cooling down at 78 K or grinding, and the green‐emissive samples can be transformed back into the blue‐emissive ones after exposure to the vapor of CH_2_Cl_2_ for 3 min or heating at 180 °C for 5 s. In addition, crystals‐C can be transformed to crystals‐B after heating at 180 °C for 5 min. As a consequence, the emission colors of **5FDPA** in the solid states can be reversibly tuned. Finally, **5FDPP** was successfully utilized to fabricate OLETs, for which the external quantum efficiency (EQE) can reach 2.2%, which is among the highest value for single‐component OLETs with symmetric source–drain electrodes.^[^ ^20^, ^21^ ^]^

## 2 Results and Discussion

### 2.1 Synthesis of **5FDPP** and **5FDPA**

**5FDPP** and **5FDPA** were synthesized by the respective reactions of 3,9‐dibromoperylene and 2,6‐dibromoanthracene with pentafluorobenzene through C—H activation catalyzed by Pd(OAc)_2_ in the presence of tricyclohexylphosphine (PCy_3_) ligand, as outlined in Scheme 1. After recrystallization in CHCl_3_/MeOH for two times, followed by sublimation, pure **5FDPP** was obtained in 54.3% yield, while **5FDPA** was purified via sublimation under vacuum for three times with a high yield of 80.1%. It is noted that this reaction can be run at gram scale with still high yield, making both **5FDPP** and **5FDPA** possible to be used as organic optoelectronic materials.

### 2.2 Crystal Structures and Photophysical Properties of **5FDPP** and **5FDPA**

**Figure 1:**
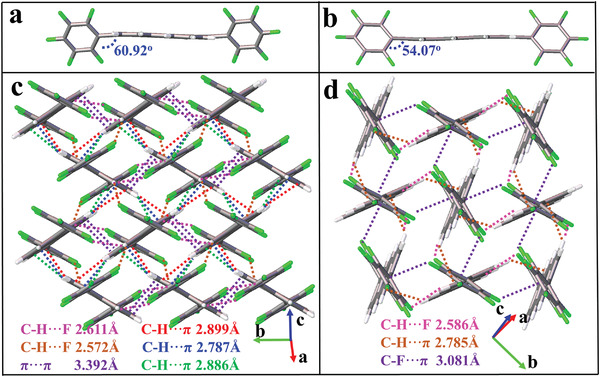
Molecular configurations and intermolecular interactions and packing modes for a,c) **5FDPP** and b,d) crystals‐B of **5FDPA**.

Single crystals of **5FDPP** were obtained by the physical vapor transport (PVT) method, and the crystal structure of **5FDPP** was successfully determined. As shown in **Figure** **1**a, **5FDPP** exhibits a twisting structure, and the dihedral angle between pentafluorophenyl group and perylene is 60.92°. Multiple C—H···*π* (2.899, 2.787, and 2.886 Å), C—H···F (2.611 and 2.572 Å), and *π*···*π* (3.392 Å) interactions were observed in the crystal lattice of **5FDPP** (Figure 1c). Besides, the neighboring molecules are packed in herringbone mode, which can form 2D transporting channels and thus facilitate carrier transporting.^[^ ^4^, ^24^ ^]^

**5FDPA** exhibits polymorphs, and three crystal phases were successfully obtained as shown in Figure S2 (Supporting Information). Figure 1b,d shows the molecular configuration and intermolecular packing for the blue‐emissive crystals (crystals‐B), which were obtained by vacuum sublimation. The molecular framework of **5FDPA** is twisting, and perfluorophenyl and anthracene groups in **5FDPA** form a dihedral angle of 54.07°. Molecules of **5FDPA** are packed in herringbone mode with multiple C—H···*π* (2.785 Å), C—F···*π* (3.081 Å), and C—H···F (2.586 Å) interactions. Dissolution of crystals‐B in chloroform solution, followed by slow evaporation of chloroform, yielded another blue‐emissive crystal forms of **5FDPA** (referred to as crystals‐C). As shown in Figure S2b,f (Supporting Information), **5FDPA** molecules in crystals‐C are also twisting with a dihedral angle of 44.77°. Comparing with crystals‐B, the intermolecular packing is obviously varied. Intermolecular short C···C contacts (3.285 Å) are observable besides C—H···F (2.629 Å) and C—F···F (2.934 Å) interactions, but neighboring molecules of **5FDPA** are not parallel. Interestingly, crystal‐to‐crystal conversion was observed for crystals‐B, which were transformed into crystals‐G after cooling at 78 K for 10 s. As shown in Figure S2c (Supporting Information), the dihedral angle of crystals‐G between the anthracene and perfluorophenyl groups is reduced to 46.77°. Anthracene units of neighboring molecules of **5FDPA** are parallel with a *π*···*π* stacking distance of 3.455 Å. Additionally, molecules are packed with C—H···F (2.444 and 2.663 Å) and C—F···F (2.918 Å) interactions (see Figure S2g in the Supporting Information). Furthermore, the crystal‐to‐crystal conversion also occurred in crystals‐G, which were transformed into blue‐emissive crystals after heating at 180 °C for 5 s (see Figure S3 in the Supporting Information). Crystal structural analysis shows that the molecular conformation and intermolecular packing for the blue‐emissive crystals that are derived from crystals‐G are almost the same as those of crystals‐B (referred to as crystals‐B′; see Figure S2d,h in the Supporting Information); the intermolecular packing adopts the herringbone mode with C—H···*π* (2.797 Å), C—F···*π* (3.056 Å), and C—H···F (2.584 Å) interactions again instead the intermolecular *π*–*π* stacking observed for crystals‐G. On the basis of X‐ray diffraction (XRD) data (see Figure S4 in the Supporting Information), crystals‐C can be transformed into crystals‐B after heating at 180 °C for 5 min. Crystals‐C cannot be transformed into crystals‐G directly when cooling it at 78 K, but crystals‐C can be converted to crystals‐B first by heating at 180 °C for 5 min, followed by cooling them at 78 K for 10 s to give crystals‐G, as shown in Figure S4 (Supporting Information). Although intercoversions among emissive polymorphs were reported,^[^ ^25^ ^]^ examples of highly emissive crystal‐to‐crystal transformations are still limited.

**Figure 2:**
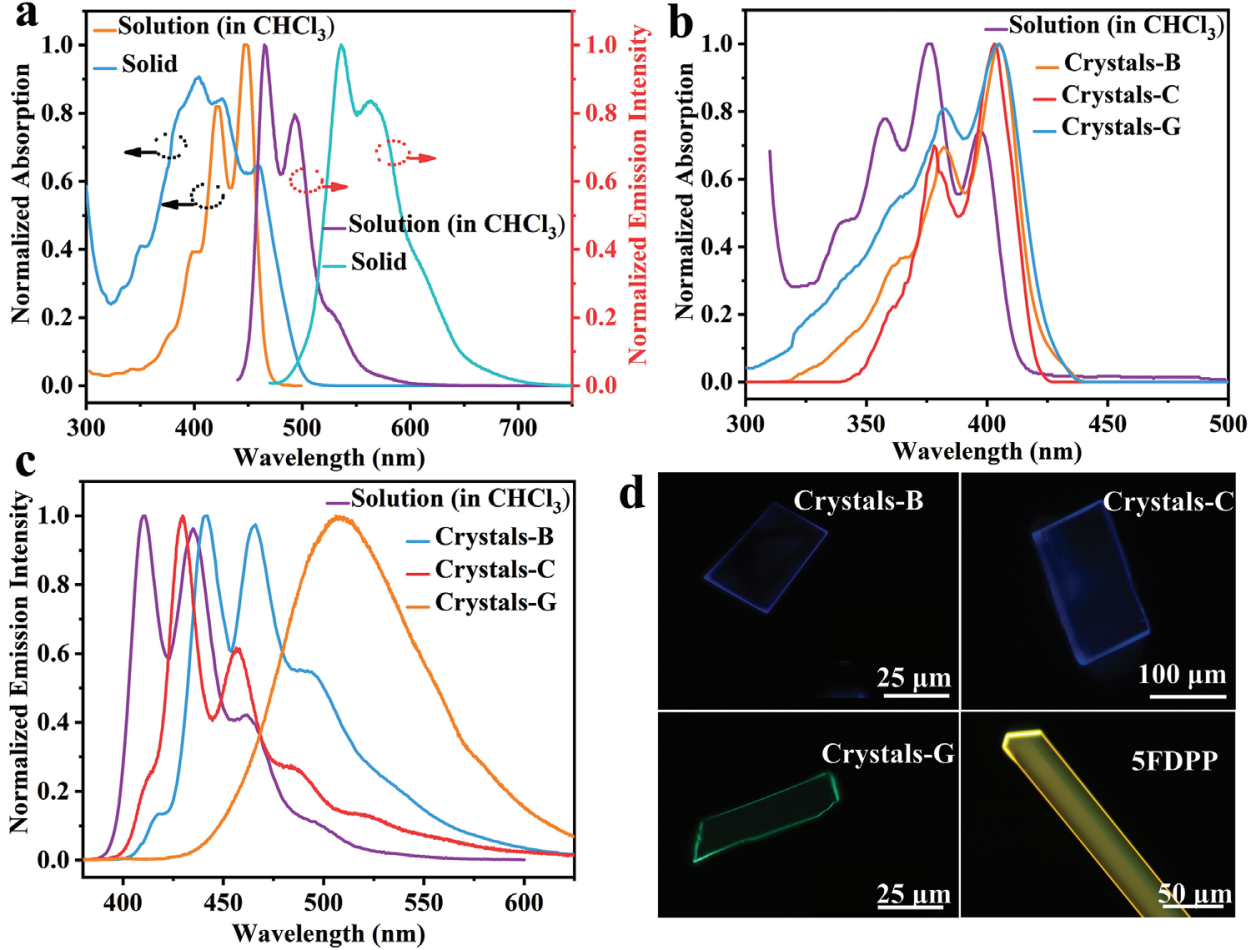
a) The absorption and photoluminescence spectra of **5FDPP** in CHCl_3_ solution and crystal state. b) The absorption spectra of **5FDPA** in CHCl_3_ solution and three crystal forms. c) The photoluminescence spectra of **5FDPA** in CHCl_3_ solution and three crystal forms. d) The fluorescence images of crystals under UV light of 365 nm.

As depicted in **Figure** **2**a, **5FDPP** absorbs strongly at 398, 422, and 447 nm in chloroform solution and emits at 466, 494, and 528 nm with a PLQY of nearly 100% and a fluorescence lifetime of 4.25 ns (see Figure 2a and also Figure S5 in the Supporting Information). The absorption peaks of the crystalline sample are redshifted to 404, 426, and 462 nm (see Figure 2a), while the respective emissions are bathochromically shifted to 537, 565, and 620 nm with a PLQY of 55% and a fluorescence lifetime of 6.79 ns (see Figure 2a and also Figure S5 in the Supporting Information).

**5FDPA** shows absorptions at around 334, 350, 369, and 390 nm in chloroform solution and emissions at around 410, 435, 461, and 497 nm (see Figure 2b,c) with a quantum yield of 16.2% and a fluorescence lifetime of 2.55 ns (see Figure S6 in the Supporting Information). In comparison, the absorptions of crystals‐B, crystals‐C, and crystals‐G are redshifted to different degrees as shown in Figure 2b. Figure 2c shows the photoluminescence spectra of **5FDPA** in solution and three crystal forms. Crystals‐B are strongly blue emissive at 440 and 466 nm with a quantum yield of 52% and an average fluorescence lifetime of 2.01 ns (see Figure S6 in the Supporting Information). Similarly, crystals‐C show blue emissions at 429 and 457 nm with a PLQY of 43.8% and an average fluorescence lifetime of 0.53 ns (see Figure S6 in the Supporting Information). But, crystals‐G become green emissive at around 508 nm exhibiting a PLQY of 62.5% and an average fluorescence lifetime of 21.17 ns (see Figure S6 in the Supporting Information).

**Figure 3:**
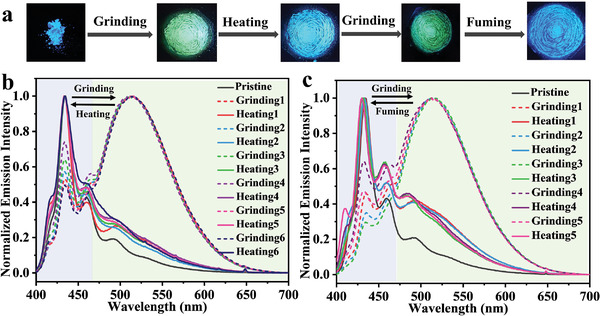
a) The corresponding photographs showing reversible emission color change under UV light (365 nm). Normalized emission spectra changes: b) grinding and heating, and c) grinding and fuming with dichloromethane.

Interestingly, the blue‐emissive crystals‐B can be converted to the green‐emissive solids, which exhibit strong emission at around 513 nm and weak emission at around 434 nm, after grinding, as shown in **Figure** **3**. The green‐emissive solids can become blue emissive after exposure to the vapors of CH_2_Cl_2_ for 3 min or heating at 180 °C for 5 s as depicted in Figure 3. Figure S9 (Supporting Information) shows the XRD patterns of the grinding sample and those of crystals‐B, crystals‐C, and crystals‐G. Clearly, XRD signals of the grinding sample contain those of crystals‐B, crystals‐C, and crystals‐G. Thus, crystals‐B were partially transformed into crystals‐C and crystals‐G after grinding. Then, it is understandable that the grinding sample shows emissions at around 513 and 434 nm. Furthermore, considering the fact that there are spectral overlaps between the absorption spectrum of crystals‐G and the emission spectra of crystals‐B and crystals‐C (see Figure S6b in the Supporting Information), förster‐resonance energy transfer (FRET) can occur between crystals‐B/crystals‐C and crystals‐G, thus leading to green emission for the grinding sample.

Alternatively, the green‐emissive grinding solid can be switched back to the blue‐emissive sample after treatment with the vapor of CH_2_Cl_2_ or heating. By comparing with the XRD patterns of crystals‐B, crystals‐C, and crystals‐G, the XRD signals attributed to those of crystals‐G disappeared, whereas those of crystals‐B and crystals‐C became more intensive under the vapor of CH_2_Cl_2_ or heating (see Figure S10 in the Supporting Information). Consequently, this agrees well with the emission color tuning from green to blue.

### 2.3 Organic field‐effect transistors (OFET) Performances of **5FDPP** and **5FDPA**

**Figure 4:**
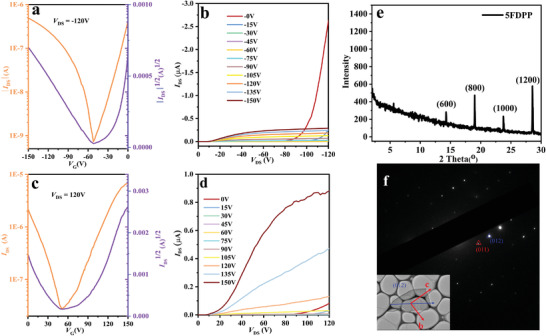
a,c) The typical transfer and b,d) output curves of **5FDPP**‐based OFETs under a,b) p‐channel and c,d) n‐channel operation modes. e) The XRD pattern of the single crystal of **5FDPP**. f) Transmission electron microscopy (TEM) image of **5FDPP** and its corresponding SAED pattern.

First, we fabricated bottom gate–top contact (BGTC) field‐effect transistors (FETs) with crystals of **5FDPP** to evaluate the charge‐transporting property, and the device fabrication details are provided in the Supporting Information. Briefly, microcrystal of **5FDPP** prepared by microspacing in air sublimation^[^ ^26^ ^]^ was carefully transferred to polymethyl methacrylate (PMMA, with a thickness of ≈20 nm; see Figure S15 in the Supporting Information) modified SiO_2_/Si substrate, followed by deposition of Ag as source–drain electrodes. **Figure** **4** shows the typical transfer and output curves under p‐channel (Figure 4a,b) and n‐channel (Figure 4c,d) operation modes. Remarkably, **5FDPP** exhibits the ambipolar semiconducting property based on the transfer and output curves, and the maximum hole and electron mobility values can reach 0.12 and 1.89 cm^2^ V^−1^ s^−1^, respectively (see Figure S16 in the Supporting Information), although the p‐channel and n‐channel charge transporting are not well balanced. On the basis of the XRD and selective area electron diffraction (SAED) data (see Figure 4e,f), the transporting direction is along the (012) plane.

Second, we investigated the charge‐transporting property of **5FDPA** by fabricating the FETs with their thin film and crystals separately. BGTC FETs were fabricated by vacuum deposition of crystalline thin films of **5FDPA** on *n‐*octadecyltrichlorosilane (OTS)‐modified SiO_2_/Si substrate, followed by deposition of Ag electrodes with a mask (for details, see the Supporting Information). The XRD pattern of the deposited thin film of **5FDPA** was measured, and the XRD signals match well with those of crystals‐B, as depicted in Figure S12 (Supporting Information). Thus, the deposited crystalline thin film is composed of microcrystals‐B. Based on the transfer and output curves (see Figure S11 in the Supporting Information), thin films of **5FDPA** show typical n‐type semiconducting behavior. The maximum electron mobility can reach 1.57 cm^2^ V^−1^ s^−1^ with the average mobility of 0.95 cm^2^ V^−1^ s^−1^ and an *I*_on_/*I*_off_ ratio of 2.5 × 10^6^ based on 27 devices (see Figure S13 in the Supporting Information).

**Figure 5:**
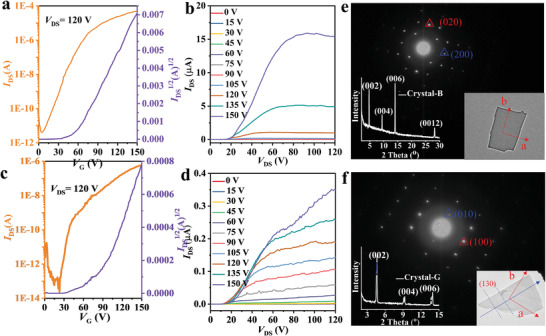
a,c) The typical transfer and b,d) output curves of a,b) crystals‐B and c,d) crystals‐G. TEM image of single crystal and its corresponding SAED pattern of e) crystals‐B and f) crystals‐G. The inset shows the XRD pattern of the single crystal.

We also utilized individual microcrystals‐B to fabricate FETs, and the device fabrication details are the same as that of **5FDPP**. Microplates of crystals‐G were yielded by cooling microcrystals‐B at 78 K for 10 s, and the FETs with microplates of crystals‐G were constructed similarly. **Figure** **5** shows the typical transfer and output curves of FETs with microplates of crystals‐B and crystals‐G. Clearly, crystals‐B exhibit n‐type transporting property. Based on XRD and SAED analyses of microplates of crystals‐B, the charge‐transporting path is along the (010) direction. The electron mobility can reach 2.65 cm^2^ V^−1^ s^−1^ with the average electron mobility of 1.04 cm^2^ V^−1^ s^−1^ and an *I*_on_/*I*_off_ ratio of 2 × 10^7^, which is the highest value among the emissive n‐type semiconductors according to the best of our knowledge (see Figure S20 in the Supporting Information). Similarly, microplates of crystals‐G also exhibit n‐type semiconducting property with the highest electron mobility of 0.17 cm^2^ V^−1^ s^−1^ along (130) direction and the *I*_on_/*I*_off_ ratio of up to 6 × 10^6^. The results reveal that crystals‐B show higher electron mobility than crystals‐G. It is noted that relatively large contact resistances of the FET devices were observed with both crystals‐B and crystals‐G. This is likely caused by relatively thick microplates of crystals‐B and crystals‐G, which show a thickness of ≈80 nm (see Figure S19 in the Supporting Information).

Third, we discussed the role of perfluorophenyl groups in switching the typical p‐type semiconducting property of perylene and anthracene into ambipolar and even n‐type transporting behaviors for **5FDPP** and **5FDPA**, respectively. On the basis of ultraviolet photoelectron spectroscopy (UPS) data of the thin film of **5FDPA** as shown in Figure S22a (Supporting Information), the highest occupied molecular orbital (HOMO) energy of the crystalline thin film of **5FDPA** was calculated to be −7.2 eV. Thus, the introduction of perfluorophenyl groups can largely lower the HOMO energy. The onset absorption of the thin film of **5FDPA** appears at 417 nm as shown in Figure S7 (Supporting Information), and thus the optical bandgap was estimated to be 2.97 eV. Consequently, the lowest unoccupied molecular orbital (LUMO) energy of **5FDPA** was calculated to be −4.23 eV. Accordingly, it is understandable that thin films of **5FDPA** exhibit n‐type semiconducting behavior. It is noted that n‐type organic semiconductors with perfluorophenyl groups were reported previously.^[^ ^22a,c^ ^]^

In comparison, the HOMO energy of **5FDPP** was measured to be −6.61 eV on the basis of UPS data of thin film (see Figure S22b in the Supporting Information). Clearly, the HOMO energy level of **5FDPP** is about 0.6 eV higher than that of **5FDPA**. Thus, the injection of holes into **5FDPP** becomes more favorable by comparing with **5FDPA**. In combination with the optical gap of **5FDPP** (2.48 eV based on the onset absorption of **5FDPP**), the LUMO energy level of **5FDPP** was calculated to be −4.13 eV. Accordingly, electron transporting is also favorable for **5FDPP** as well as **5FDPA**. Therefore, these results agree well with the observation that **5FDPP** shows the ambipolar semiconducting property, but the hole and electron transporting properties are not balanced.

**Figure 6:**
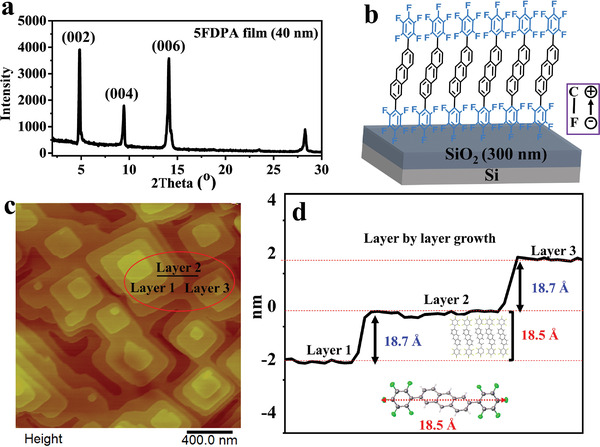
a) XRD pattern of the deposited thin film of **5FDPA**. b) Schematic diagram of the vertical alignment of **5FDPA** on the OTS‐modified SiO_2_/Si substrate. c) AFM height image of the deposited thin film of **5FDPA**. d) The step height of layer‐by‐layer growth of **5FDPA** on the OTS‐modified SiO_2_/Si substrate. The inset shows the molecular length of **5FDPA** in crystals‐B.

Alternatively, on the basis of the XRD pattern of crystalline thin film of **5FDPA** (see **Figure** **6**a), which was deposited on the OTS‐modified SiO_2_/Si substrate, the sharp and intensive (*00l*) diffraction peaks indicate that molecules of **5FDPA** are perpendicularly arranged on the substrate. The *d*‐spacing based on the first‐order diffraction (4.82°) in the perpendicular direction of the substrate (along the *c*‐axis of crystals‐B) is estimated to be 18.3 Å, which is almost the same as the molecular length of **5FDPA** (18.5 Å, the molecular length across the two perfluorophenyl groups; see Figure 6d). Therefore, it can be inferred that molecules of **5FDPA** are perpendicularly packed on the substrates as illustrated in Figure 6b. Alternatively, the crystalline thin films were characterized by atomic force microscope (AFM). Figure 6c,d shows the AFM height images. Clearly, the crystalline thin films of **5FDPA** show layer‐by‐layer growth in the substrate, and the interlayer distance is 18.7 Å, which is close to the molecular length of **5FDPA** (18.5 Å). This result agrees well with the fact that molecules of **5FDPA** are perpendicularly arranged on the substrate. According to previous studies,^[^ ^27^ ^]^ such ordered orientation of perfluorophenyl groups with polar C—F bonds can lower the HOMO energy level of **5FDPA** and hole injection from electrodes becomes unfavorable, but electron injection from electrodes can be facilitated.

Similarly, crystalline thin films of **5FDPP** were also composed of layered structures according to the AFM image (see Figure S24 in the Supporting Information). The interlayer distance was measured to be 18.5 Å, which is almost the same as the length of **5FDPP** across the two perfluorophenyl groups (18.6 Å; see Figure S24 in the Supporting Information). Thus, it can be inferred that molecules of **5FDPP** are also perpendicularly grown on the substrate in a layer‐by‐layer manner. As for **5FDPA**, the ordered arrangement of perfluorophenyl groups with polar C—F bonds favors electron transporting.

Additionally, the intermolecular transfer integrals were calculated for crystals‐B and crystals‐G of **5FDPA**. As shown in Figure S25 (Supporting Information), the intermolecular transfer integral for the electron transporting is 67.1 meV along the *b*‐axis in crystals‐B of **5FDPA**. In comparison, the integrals of crystals‐G of **5FDPA** for the electron transporting along the *a*‐axis and *b*‐axis are 83.1 and 9.9 meV, respectively. These calculation results are consistent with the observation that the electron mobility of crystals‐B along the (010) direction is higher than that of crystals‐G along the (130) direction. In the same way, the transfer integrals of **5FDPP** for hole and electron transporting were calculated to be 2.64 and 23.46 meV along the charge‐transporting plane (see Figure S26 in the Supporting Information). This is consistent with the experimental results that the crystal of **5FDPP** shows an unbalanced ambipolar‐transporting property with higher electron mobility and lower hole mobility.

### 2.4 OLET Performances of **5FDPP** and **5FDPA**

Finally, we utilized crystals of emissive semiconductors of **5FDPA** and **5FDPP** to fabricate OLET devices with Ag as source–drain electrodes (see Figure S14 in the Supporting Information). Our preliminary results show that electroluminescence was detected near Ag electrodes when the respective OFETs with crystals‐B and crystals‐G were switched on (see Figure S27 in the Supporting Information). The collected electroluminescence spectra are almost overlapped with the respective photoluminescence spectra of crystals‐B and crystals‐G. However, the electroluminescence performances were poor, and the emission could not be tuned in the conducting channel because of the unipolar electron‐transporting property of crystals‐B and crystals‐G.

**Figure 7:**
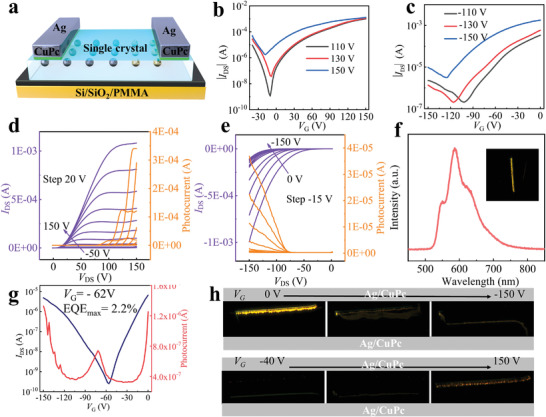
a) The schematic diagram of OLET devices with CuPc insertion between Ag electrode and semiconductor layer. b) The transfer curves of **5FDPP**‐based OLET devices under positive voltages. c) The transfer curves of **5FDPP**‐based OLET devices under negative voltages. d) The output curves of **5FDPP**‐based OLET devices for n‐channel. e) The output curves of **5FDPP**‐based OLET devices for p‐channel. f) The electroluminescence spectra of **5FDPP**‐based OLET devices. g) The photocurrent versus *V*_G_ for **5FDPP**‐based OLET devices without insertion of CuPc. h) The spatially controlled emission image of the **5FDPP**‐based OLET devices by varying *V*_G_.

In comparison, the ambipolar charge‐transporting property of **5FDPP** enables the injection and transporting of both electron and hole carriers. To our delight, electroluminescence was observed between the source and drain Ag electrodes (without modification) when the field‐effect transistors were switched on under either positive (n‐channel) or negative (p‐channel) gate voltages. The EQE can reach 2.2% as shown in **Figure** **7**g, which is among the highest value for single‐component OLETs, to the best of our knowledge. Nevertheless, the unbalanced charge injection and transporting caused the *I*_DS_ of devices to be low (see Figure S28 in the Supporting Information). To enhance the hole injection, we deposited a thin layer (≈20 nm) of CuPc as the excellent charge‐transporting material^[^ ^28^ ^]^ between Ag electrodes and the crystal of **5FDPP**. The device structure is shown in Figure 7a. The resulting device shows high *I*_DS_ after the Ag electrodes were modified by CuPc as shown in Figure 7, where the transfer curves and the output curves for n‐channel and p‐channel, respectively, are displayed. The OLETs with the crystal of **5FDPP** show much brighter electroluminescence, and the electroluminescence spectrum with emission peaks at 548, 586, and 629 nm, which matches well with the photoluminescence spectrum of **5FDPP** crystal (see Figures 2a and 7f). As shown in Figure S29 (Supporting Information), the emitting light brightness as a function of gate voltage gives a maximum brightness of 750 cd m^−2^, by assuming that the width of the emission zone is 2 µm, which is commonly accepted.^[^ ^29^ ^]^ In addition, the current density is up to 145 kA cm^−2^ for **5FDPP**‐based OLET devices by assuming the current flow to be confined at one molecular layer.^[^ ^30^ ^]^ Furthermore, the emission zone in the conducting channel can be spatially controlled between the source and drain electrodes, as shown in Figure 7h, under either p‐channel or n‐channel operation modes. The emission images in Figure 7h were recorded by charged coupled device (CCD) during the operation in real time, and the movement of the emission zone can reduce the loss of excitons.

## 3 Conclusion

In conclusion, we present new emissive ambipolar and even n‐type organic semiconductors by linking perfluorophenyl groups with polycyclic aromatic hydrocarbons. **5FDPP** and **5FDPA** were synthesized in one step via C—H activation in high yields. The results show that i) **5FDPP** exhibits the ambipolar semiconducting property with hole and electron mobilities up to 0.12 and 1.89 cm^2^ V^−1^ s^−1^, and an emission quantum yield of 55%, and ii) **5FDPA** behaves as an emissive n‐type semiconductor with polymorphs exhibiting high fluorescence quantum yield and electron mobility. The possible role of perfluorophenyl groups was explored in switching the p‐type semiconducting property of perylene and anthracene into ambipolar and n‐type semiconductors for **5FDPP** and **5FDPA**, respectively.

Three crystalline forms of **5FDPA**, referred to as crystals‐B, crystals‐C, and crystals‐G, were obtained by vacuum sublimation, crystallization from CHCl_3_, and cooling at 78 K, respectively. Crystals‐B and crystals‐C are blue emissive with emission peaking at around 440 and 429 nm with fluorescence quantum yields of 52% and 43.8%, respectively. In comparison, crystals‐G are green emissive at around 508 nm with a fluorescence quantum yield of 62.5%. Notably, crystals‐B exhibit remarkably a high electron mobility of up to 2.65 cm^2^ V^−1^ s^−1^, which is the highest electron mobility for n‐type organic semiconductors with high fluorescence quantum yields.

Furthermore, we successfully fabricated organic light‐emitting transistors with crystals of **5FDPP** by using Ag as source–drain electrodes. The devices exhibited spatially controlled electroluminescence in the transporting channels with an EQE of up to 2.2%, which is the highest value for single‐component OLETs with symmetric electrodes. In addition, the OLET devices exhibit a high current density of 145 kA cm^−2^, which can be attributed to the inherent high carrier transport and strong emission properties of **5FDPP**.

## 4 Experimental Section

### Synthesis of **5FDPP**

To a Schlenk tube (100 mL) equipped with a magnetic stir bar, 3.9‐dibromoperylene (1 g, 2.44 mmol), pentafluorobenzene (1.64 g, 9.77 mmol), Pd(OAc)_2_ (109 mg, 0.5 mmol), PCy_3_ (280 mg, 1 mmol), cesium carbonate (Cs_2_CO_3_, 954 mg, 2.93 mmol), and toluene (30 mL) were added. The mixture was degassed and charged with nitrogen for three times. The mixture was heated at 120 °C (oil bath) for 14 h. After cooling to room temperature, the toluene was removed under reduced pressure. The residue was purified by column chromatography with petroleum ether (b.p. = 60–90 °C) as the eluent to give a crude product. The crude product was recrystallized in CHCl_3_/MeOH for two times to give the product, and the resulting product was further purified by sublimating at 260 °C under the vacuum of 10^−1^ Pa to give a yellow solid of **5FDPP** (773 mg, 54.3% yield).

^1^H NMR (700 MHz, C_2_D_2_Cl_4_, 373 K) *δ* (ppm): 8.31–8.29 (m, 4H), 7.54 (dd, *J* = 8.3, 7.4 Hz, 2H), 7.47 (d, *J* = 7.6 Hz, 2H), 7.36 (d, *J* = 8.4 Hz, 2H); ^13^C NMR (176 MHz, C_2_D_2_Cl_4_, 298 K) *δ* (ppm): 144.8, 141.2, 138.0, 132.8, 132.7, 131.0, 130.0, 128.7, 127.8, 125.3, 123.6, 121.6, 120.4, 114.4; ^19^F NMR (659 MHz, C_2_D_2_Cl_4_, 298 K) *δ* (ppm): −138.69 (dd, *J* = 22.9, 8.2 Hz, 4F), −154.06 (t, *J* = 20.4 Hz, 2F), −161.35 (td, *J* = 21.7, 7.9 Hz, 4F); HRMS (High Resolution Mass Spectrometry) (Matrix‐Assisted Laser Desorption Ionization, MALDI) *m*/*z*: [M]^+^ calcd. for C_32_H_10_F_10_ 584.0617; found 584.0622; anal. calcd. for C_32_H_10_F_10_: C, 65.77; H, 1.72; found: C, 65.38; H: 1.75.

### Synthesis of **5FDPA**

To a Schlenk tube (100 mL) equipped with a magnetic stir bar, 2,6‐dibromoanthracene (1.2 g, 3.57 mmol), pentafluorobenzene (2.4 g, 14.3 mmol), Pd(OAc)_2_ (160 mg, 0.71 mmol), PCy_3_ (400 mg, 1.42 mmol), Cs_2_CO_3_ (1.4 g, 4.28 mmol), and toluene (30 mL) were added. The mixture was degassed and charged with nitrogen for three times. The mixture was heated at 120 °C (oil bath) for 12 h. After cooling to room temperature, the mixture was poured into MeOH (100 mL) at room temperature, and the resulting mixture was filtered and washed with H_2_O, MeOH, and *n‐*hexane. The crude product was then purified by sublimating three times at 220 °C under the vacuum of 10^−1^ Pa to give a white solid of **5FDPA** (1.466 g, 80.1% yield).

^1^H NMR (500 MHz, C_2_D_2_Cl_4_, *δ*): 8.59 (s, 2H), 8.20–8.19 (m, 4H), 7.57 (dd, *J* = 8.9, 1.6 Hz, 2H); ^13^C NMR (176 MHz, C_2_D_2_Cl_4_, *δ*): 144.6, 140.8, 138.1, 131.9, 131.6, 130.6, 128.9, 127.2, 126.9, 124.2, 116.0; ^19^F NMR (659 MHz, C_2_D_2_Cl_4_, *δ*): −142.09 (dd, *J* = 19.8, 13.2 Hz, 4F), −154.41 (t, *J* = 19.8 Hz, 2F), −161.27 (dt, *J* = 19.8, 13.2 Hz, 4F); HRMS (MALDI) *m*/*z*: [M]^+^ calcd. for C_26_H_8_F_10_, 510.0461; found 510.0460; anal. calcd. for C_26_H_8_F_10_: C, 61.19; H, 1.58; found: C, 61.20; H: 1.58.

## Conflict of Interest

The authors declare no conflict of interest.

## Supporting information

Supporting Information

## Data Availability

The data that support the findings of this study are available in the supplementary material of this article.
