# Supplementary material for: High‐Performance Ambipolar and n‐Type Emissive Semiconductors Based on Perfluorophenyl‐Substituted Perylene and Anthracene
Source: Adv Sci (Weinh). 2023 Mar 26;10(15):2300530. doi: 10.1002/advs.202300530 (PMC10214240; doi:10.1002/advs.202300530)
Supplement: Supplementary file 1 — Supporting Information [file ADVS-10-2300530-s001.pdf]

# Supporting Information

**High-Performance *n*-type and ambipolar emissive semiconductors based on perfluorophenyl-substituted perylene and anthracene**

*Liangliang Chen,*[a,b,#] *Zhengsheng Qin*[a,b,#]*, Han Huang,*[c] *Jing Zhang,*[a,b] *Zheng Yin,*[a,b] *Xiaobo Yu,*[a,b] *Xi-sha Zhang,*[a,b] *Cheng Li,*[a] *Guanxin Zhang,*[a] *Miaofei Huang,*[a,b] *Huanli Dong*[a]*, Yuanping Yi,*[a] *Lang Jiang,*[a] *Hongbing Fu,*[c] *Deqing Zhang*[a,b]*

[a] Beijing National Laboratory for Molecular Sciences, CAS Key Laboratory of Organic Solids, Institute of Chemistry, Chinese Academy of Sciences, Beijing 100190, P. R. China;

[b] University of Chinese Academy of Sciences, Beijing 100049, P. R. China;

[c] Beijing Key Laboratory for Optical Materials and Photonic Devices, Department of Chemistry, Capital Normal University, Beijing, 100048, China.

# Contents

**1. Materials** ........ **2**
**2. Characterization techniques** ........ **2**
**3. OFET characterization** ........ **3**
3.1 The device of vacuum-deposited film ........ 3
3.2 The device of single crystals ........ 3
**4. OLETs characterization** ........ **5**
**5. The polymorphs of 5FDPA and SCSC (single crystal to single crystal) transformation** ........ **7**
**6. The photophysical properties** ........ **9**
**7. Performance of OFETs with vacuum-deposited films of 5FDPA** ........ **12**
**8. Performance of OFETs with crystals of 5FDPP and 5FDPA** ........ **14**
**9. The characterizations of energy levels of 5FDPA and 5FDPP** ........ **18**
**10. The calculated transfer integrals** ........ **20**
**11. Performance of OLETs with crystals of 5FDPP and 5FDPA** ........ **21**
**12. Crystallographic data** ........ **24**
**13. $^{1}$H NMR, $^{13}$C NMR and $^{19}$F NMR spectra** ........ **30**
**14. References** ........ **34**

## 1. Materials

The reagents and starting materials were commercially available and used as received otherwise specified elsewhere. 2,6-Dibromoanthracene was purchased from TCI. 3,9-Dibromoperylene was purchased from Shanghai Macklin Biochemical Co., Ltd. Pentafluorobenzene was purchased from J&K Scientific Ltd.

## 2. Characterization techniques

$^{1}$H NMR and $^{13}$C NMR and $^{19}$F NMR spectra were measured with Bruker AVANCE III 500 MHz and Bruker AVANCE NEO 700 MHz spectrometers. High-resolution mass spectral (HRMS) data were collected on either 9.4T Solarix Mass instrument and the mass analyzers are MALDI-FTICR (Fourier transform ion cyclotron resonance). Elemental analyses (EA) were performed on a Carlo-Erba-1106 instrument.

Absorption spectra were recorded on the HITACHI UH4150 spectrophotometer. Emission spectra and lifetime were recorded on the Hitachi FP-6000 spectrometer and Edinburgh FLS980. Photoluminescence quantum yield of solution and solid were measured on the HAAMATSU C11347.

Atomic force microscopy (AFM) images were recorded using a Digital Instruments Nanoscope IIIa multimode atomic force microscope in tapping mode under ambient conditions. Two-dimensional grazing-incidence wide-angle X-ray scattering (GIWAXS) measurements were conducted on a Xenocs SAXS/WAXS system with X-ray wavelength of 1.54 Å; the film samples were irradiated at a fixed angle of 0.2$^{o}$. The thickness of the film for AFM and GIWAXS measurement was about 40 nm, which was deposited under the vacuum ($10^{-6}$ mbar) at the rate of 0.1~0.2 Å/s. The UPS measurement was performed on an Axis Ultra DLD (Kratos, UK) spectrometer with an unfiltered He I (21.22 eV) excitation source. The thickness of the film for UPS measurement was about 15 nm, which was deposited under the vacuum ($10^{-6}$ mbar) at the rate of 0.1Å/s. XRD measurements were conducted on a PANalytical Empyrean with X-ray wavelength of 1.54 Å.

The diffraction data for the single crystals were collected with a Rigaku Saturn diffractometer with CCD area detector with Cu at 170 K or 110 K. **5FDPP** were obtained by physical vapor transport (PVT) method heating at 260 $^{o}$C in the high-temperature zone. Crystals-B of **5FDPA** were obtained by physical vapor transport (PVT) method heating at 220 $^{o}$C in the high-temperature zone. Crystals-C was obtained by recrystallization of **5FDPA** in chloroform solution. Crystals-G was obtained by cooling crystals-B with liquid nitrogen for 10 s, followed by warming the crystals to room temperature. Crystals-B′ was obtained by heating crystals-G at 180 $^{o}$C for 5 s, followed by cooling to room temperature. Crystallographic data reported in this paper were deposited in the Cambridge Crystallographic Data Centre (CCDC No. 2164674 for **crystals-C**; CCDC No. 2164676 for **crystals-G**; CCDC No. 2164677 for **crystals-B′**; and CCDC No.2164678 for **crystals-B**; CCDC No. 2178552 for **5FDPP**;).

**Computational Details:** The properties of ground state and ionic states of

those molecules were studied based on the optimized structure at the ωB97XD/def2SVP level. All dimers are extracted from the experimental crystal structures without optimization. The charge transfer integrals of dimers were performed at the ωB97XD/def2SVP level. All the calculations were carried out with the Gaussian 16 package

## 3. OFET characterization

### 3.1 The device of vacuum-deposited film.

The substrates were firstly cleaned by sonication in acetone and water and immersed in Piranha solution (2:1 mixture of sulfuric acid and 30% hydrogen peroxide) and heated to 100 $^{o}$C for 30 min, followed by rinsing with deionized water and isopropyl alcohol for several times, and they were blow-dried with nitrogen. Then, the substrate was processed by UV ozone for about 10 min. After that, the substrates were placed into a petri dish, and one drop of *n*-octadecyltrichlorosilane (OTS) was dropped into the middle of the petri dish. The system was stored under vacuum at 125 $^{o}$C for 4 h to form an OTS self-assembled monolayer. After the substrate surfaces were modified with OTS, they were washed with n-hexane, $CHCl_3$ and isopropyl alcohol sequentially. These substrates were used directly for fabrication of devices with **5FDPA**.

*Fabrication of FETs with Thin-films of* ***5FDPA***

Bottom gate bottom contact (BGTC) field-effect transistors were fabricated to explore the semiconducting performance. **5FDPA** was slowly deposited on an OTS-treated $Si/SiO_2$ substrate under a high vacuum ($10^{-6}$ mbar) as the active layer with the thickness of 40 nm at the rate of 0.1~0.2 Å/s. Then, an interdigital mask with a channel length of 7140 um and a channel width of 270 um was used. Ag electrode was deposited to the surface with a thickness of 50 nm. After peeling the mask, the BGTC devices were fabricated and used for measurement directly. All these processes were carried out in the glove box.

### 3.2 The device of single crystals

The substrates were firstly cleaned by sonication in acetone and water and immersed in Piranha solution (2:1 mixture of sulfuric acid and 30% hydrogen peroxide) and heated to 100 $^{o}$C for 30 min, followed by rinsing with deionized water and isopropyl alcohol for several times, and they were blow-dried with nitrogen. Then, the substrate was heated at 80 °C for 1 h under vacuum. After that, the substrate was washed with n-hexane, $CHCl_3$ and isopropyl alcohol sequentially. A thin layer of PMMA was spin-coated onto the substrates with a speed of 5000 r/min for 50 s. The concentration of PMMA was 1% in toluene. The thickness of PMMA was about 20 nm according to the AFM height image. Then, the substrates were heated to 120 $^{o}$C for 10 min. After cooling to room temperature, these substrates were used directly for fabrication of devices with crystals of **5FDPP** and **5FDPA**.

*Fabrication of OFETs with single crystals of **5FDPP** and **5FDPA***

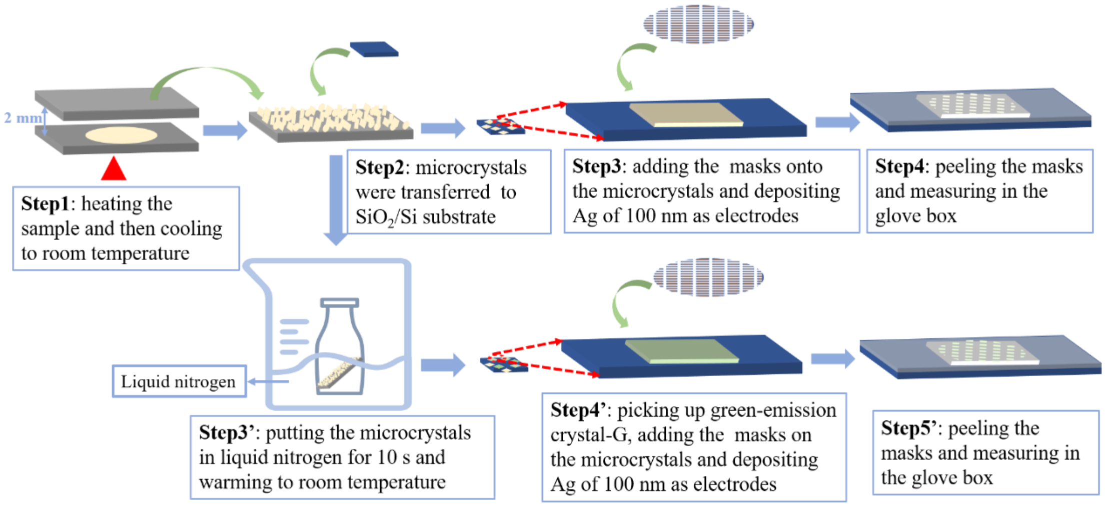


**Figure S1** Schematic diagram of the fabrication processes of OFETs with single crystals of **5FDPP** and **5FDPA**.

*Single-crystal OFETs with **5FDPP** (step1-step4)*

The micro-co-crystals were grown by micro-spacing in-air sublimation of co-crystals when heating to 280 °C for 10 min.[S1] After cooling to temperature, the microcrystals were transferred onto the substrates. The copper mesh was used as a mask to the microcrystals. Then Ag was deposited to the surface with a thickness of 100 nm as source-drain electrodes. After peeling the mask, the BGTC single-crystal field-effect transistors based on co-crystals were fabricated and used for measurement directly in the glove box.

*Single-crystal OFETs with crystals-B (step1-step4)*

The microcrystals were grown by micro-spacing in-air sublimation of **5FDPA** when heating to 235 °C for 10 min.[S1] After cooling to temperature, the microcrystals were transferred onto the substrates. The copper mesh was used as a mask to the microcrystals. Then, Ag was deposited to the surface with a thickness of 100 nm as source-drain electrodes. After peeling the mask, the BGTC single-crystal field-effect transistors with crystals-B were fabricated and used for measurement directly in the glove box.

*Single-crystal OFETs with crystals-G (step1-step5')*

The microcrystals were grown by micro-spacing in-air sublimation of **5FDPA** when heating to 235 °C for 10 min.[S1] After cooling to temperature, the microcrystals were cooled to 78 K in liquid nitrogen for 10 s. After warming to room temperature, the microcrystals were transferred onto the substrates. The green-emission microcrystals were picked up under a microscope and the copper mesh was used as a mask to the microcrystals. Then Ag was deposited to the surface with a thickness of 100 nm as source-drain electrodes. After peeling the mask, the BGTC single-crystal

field-effect transistors based on crystals-G were fabricated and used for measurement directly in the glove box.

*OFETs measurements*

Characteristics of the devices were measured in the glove box using a Keithley 4200 SCS semiconductor parameter analyzer. The mobility of the OFETs in the saturation region was extracted from the following equation:

$I_{DS}= \mu C_i(V_G-V_{Th})^2 W/2L$

Where $I_{DS}$ is the current collected by the drain electrode; $L$ and $W$ are the channel length and width, respectively; $\mu$ is the mobility of the device; $C_i$ is the capacitance per unit area of the gate dielectric layer (11.5 nF/cm$^2$ for film devices and 10.0 nF/cm$^2$ for single-crystal devices); $V_G$ is the gate voltage, and $V_{Th}$ is the threshold voltage.

## 4. OLETs characterization

*Fabrication of OLETs with single crystals of* ***5FDPP***

The Single-crystals were prepared by physical vapor transfer method under argon environment. A thin layer of PMMA was spin-coated onto Si/$SiO_2$ substrate from a PMMA solution (6 mg ml$^{-1}$; chlorobenzene as a solvent) with a speed of 5000 r/min for 50 s in the glove box and then annealed at 90°C for 2 hours. Then large sized single crystals were transferred to the PMMA treated Si/SiO2 substrate by shearing friction in the glove box. The symmetric electrodes Ag (50 nm) or CuPc (20 nm)/Ag (50 nm) were fabricated by thermal evaporation with a mask for hole injection and electron injection, respectively. The PMMA buffer layer (20 nm, see Figure S14) has a negligible effect on the capacitance of the $SiO_2$ (300 nm), and thus, the capacitance used in the calculation of the mobility of OLET is still 10 nF cm$^{-2}$.

*OLETs measurements*

The electrical characteristics measurement was conducted by PDA FS-Pro 380 under inert atmosphere and the optical image was taken by a Nikon digital camera. The electroluminescence spectra were obtained by a spectrometer made by Ocean Optics. the EQE of EL device is acquired from the number of the collection emissive photons $n_v$ divided by the number of injected carriers $n_e$ according to the following equation:

$$EQE = \frac{n_v}{n_e}$$

The photocurrent of standard light source collected by PMT can be calculated by the following equation:

$$I_{PMT} = \int P_C(\lambda)\eta(\lambda)d\lambda = \int P_C(\lambda) \cdot B \cdot \tilde{\eta}(\lambda)d\lambda$$

Where $\eta(\lambda)$ and $\tilde{\eta}(\lambda)$ are spectral response curve and normalized curve of PMT, respectively. The spectrum of a standard light source $P_C(\lambda)$ is a known value. Then, the value of B can be calculated directly according to the calculation formula of $I_{PMT}$. The $P_S(\lambda)$ of the sample light source can also be normalized to $A \cdot \tilde{P}_S(\lambda)$, where the normalized spectrum $\tilde{P}_S(\lambda)$ can be measured directly by the spectrometer.

For the sample source, photocurrent of sample source collected by PMT can be calculated by the following equation: $I_{PMT} = \int A \cdot \tilde{P}_S(\lambda) B \cdot \tilde{\eta}_S(\lambda) d\lambda$, only A is an unknown quantity, then the optical power spectrum of the sample source $P_S(\lambda)$ can be directly calculated by the formula.

Hence, the number of total photons $n_v$ from detected emissive spectra ranging from starting and ending wavelength can be calculated through mathematical integral.

$$n_v = \int \frac{p_s(\lambda)\lambda d\lambda}{hc}$$

Where λ is wavelength of the emissive light, in nm. $h$ is Planck constant ($6.626 \times 10^{-34}$ J s). $c$ is velocity of light in vacuum ($3.0 \times 10^{8}$ m $s^{-1}$). The number of injected carriers $n_e$ during the test is calculated from the recombination current $I$ at saturation region of OLET devices divided by the elementary charge e = $1.602 \times 10^{-19}$ C. Therefore, the EQE is calculated by following equation:

$$EQE = \frac{\int \frac{P_s(\lambda) d\lambda}{hc/\lambda}}{I/e}$$

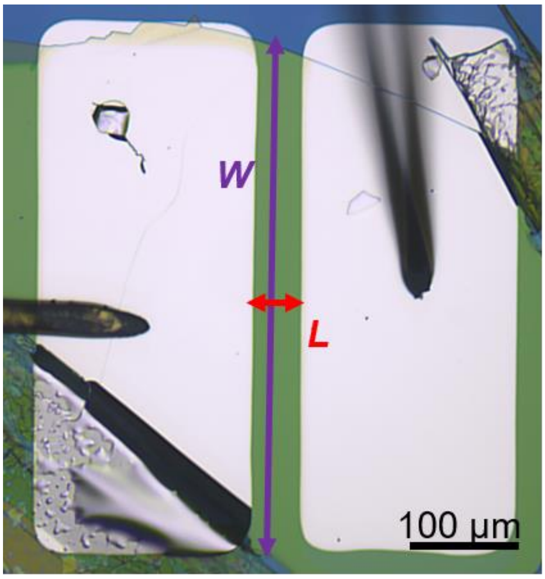


Figure S1-1 The device structure of the single-crystal OLETs; two electrodes in grey color are on the top of the crystal (in green color); *W* and *L* are the channel width and length, respectively.

## 5. The polymorphs of 5FDPA and SCSC (single crystal to single crystal) transformation

### *Crystals-B to Crystals-G transformation*

By putting blue-emissive crystals-B into liquid nitrogen for 10 s, the green-emission crystals-G were obtained. However, the conversion was not complete according to XRD patterns in Figure S4.

### *Crystals-C to crystals-B and crystals-G transformation*

Crystals-C cannot be transformed into green-emissive crystals-G directly by cooling at 78 K, but they can be converted to crystals-B firstly by heating at 180 $^{o}$C for 5 min and then cooling them at 78 K for 10 s to give green-emissive crystals-G. Obviously, the conversion of crystals-C to crystals-B was nearly complete, but crystals-B cannot be fully transformed to crystals-G according to XRD patterns in Figure S5.

### *Crystals-G to Crystals-B transformation*

When heating crystals-G to 180 $^{o}$C for 5 s, it can be converted to crystals-B completely based on the XRD patterns in Figures S4 and S5. Besides, when dissolving crystals-B or crystals-G in chloroform solution, the plate-like crystals-C were yielded by slow evaporation of chloroform.

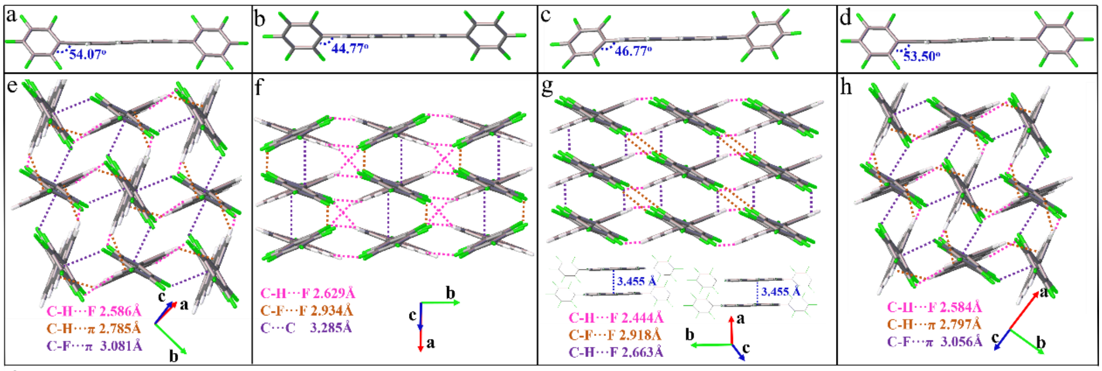


i

| State | Crystals-B | Crystals-C | Crystals-G | Crystals-B′ |
|---|---|---|---|---|
| Crystal system | monoclinic | monoclinic | triclinic | monoclinic |
| a/Å | 6.8685(3) | 22.6177(4) | 3.7710(2) | 37.783 (2) |
| b/Å | 7.4556(3) | 6.59380(10) | 6.4409(3) | 7.4314(4) |
| c/Å | 37.8682(17) | 13.3302(2) | 19.5947(9) | 6.8606(3) |
| α/° | 90 | 90 | 97.053(4)° | 90° |
| β/° | 94.708(4)° | 105.983(2) | 93.355(4)° | 95.205(2)° |
| γ/° | 90° | 90 | 90.652(4)° | 90° |
| Volume/Å$^3$ | 1932.64(14) | 1911.17(6) | 471.43(4) | 1918.38(17) |

**Figure S2** Molecular configurations and intermolecular interactions and packing modes for crystals-B (a, e), crystals-C (b, f), crystals-G (c, g) and crystals-B′ (d, h). (g) The crystallographic parameters of crystals-B, crystals-C, crystals-G and crystals-B′. Crystals-B obtained by sublimation; crystals-C obtained by recrystallization in chloroform solution; crystals-G obtained by cooling crystals-B to 78 K for 10 s; crystals-B′ obtained by heating crystals-G to 180 $^{o}$C for 5 s. Displacement ellipsoids are drawn at 50% probability level.

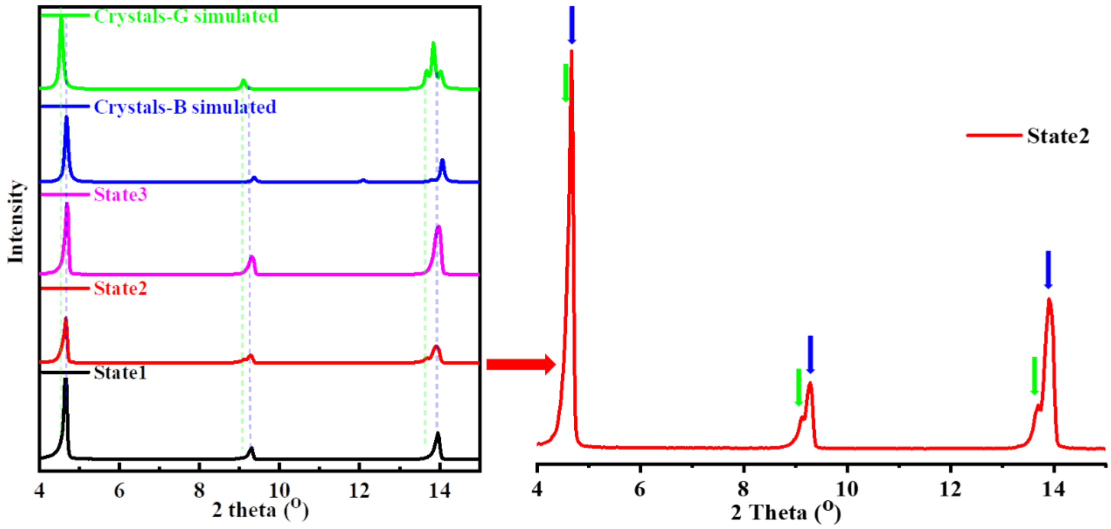


**Figure S3** The XRD patterns of the crystalline samples under different conditions. State 1: the pristine solid (belonging to crystals-B phase) obtained by sublimation (black); state 2: cooling pristine solid at 78 K for 10 s, followed by warming to temperature (red); state 3: heating the crystalline sample that had been cooled at 180 $^{o}$C for 5 s, followed by cooling to temperature (pink). The simulated XRD patterns of crystals-B (blue) and crystals-G (green).

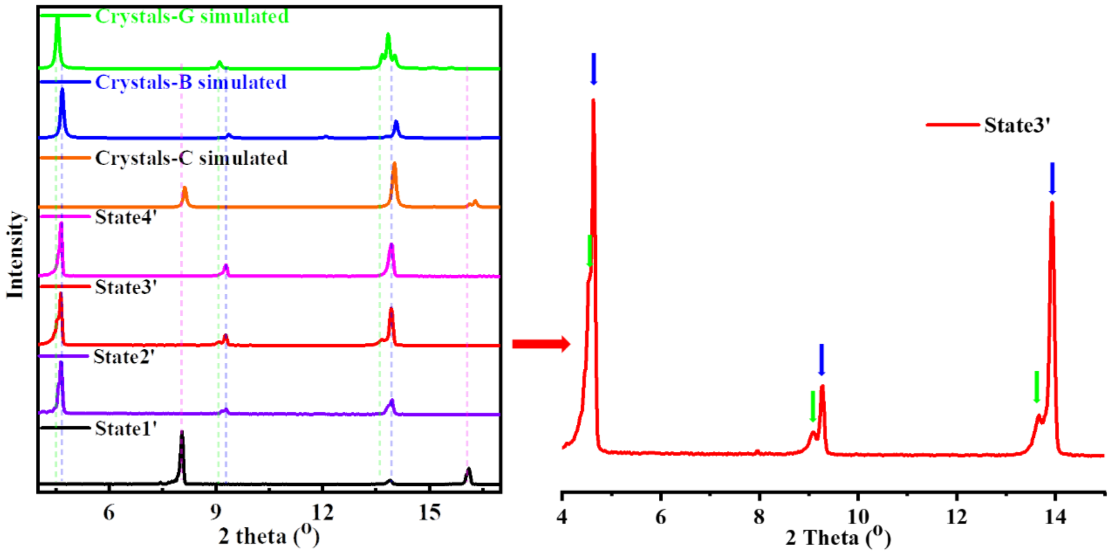


**Figure S4** The XRD patterns of the crystalline samples under different conditions. State 1′: the pristine solid (belonging to crystals-C phase) obtained by recrystallization (black); state 2′: heating the pristine solid to 180 $^{o}$C for 5 min and then cooling to room temperature (purple); state 3′: cooling the crystalline sample that had been heated at 78 K for 10 s and then warming to room temperature (red); state4′: heating the crystalline sample that had been cooled at 180 $^{o}$C for 5 s and then cooling to room temperature (pink). The simulated XRD patterns of crystals-C (orange), crystals-B (blue) and crystals-G (green).

## 6. The photophysical properties

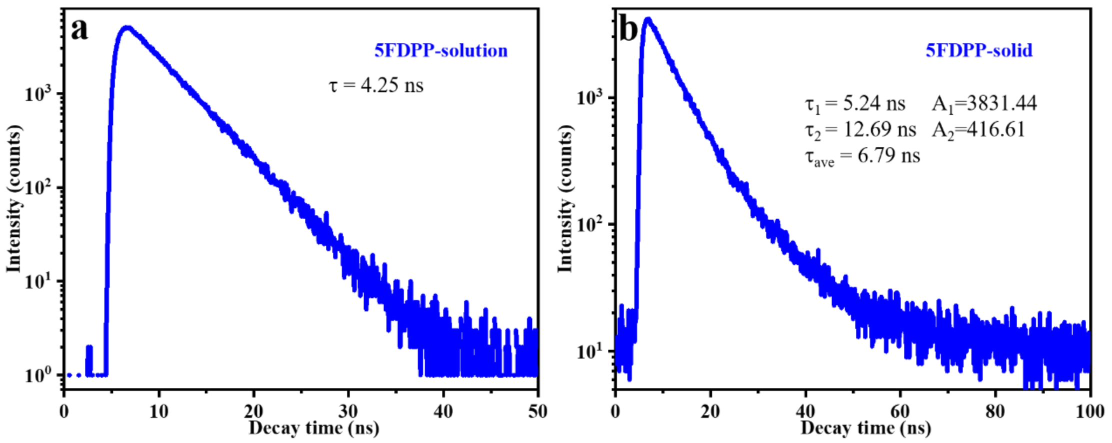


**Figure S5** The photoluminescence decays of solution in $CHCl_3$ (a) and crystal (b) of **5FDPP**.

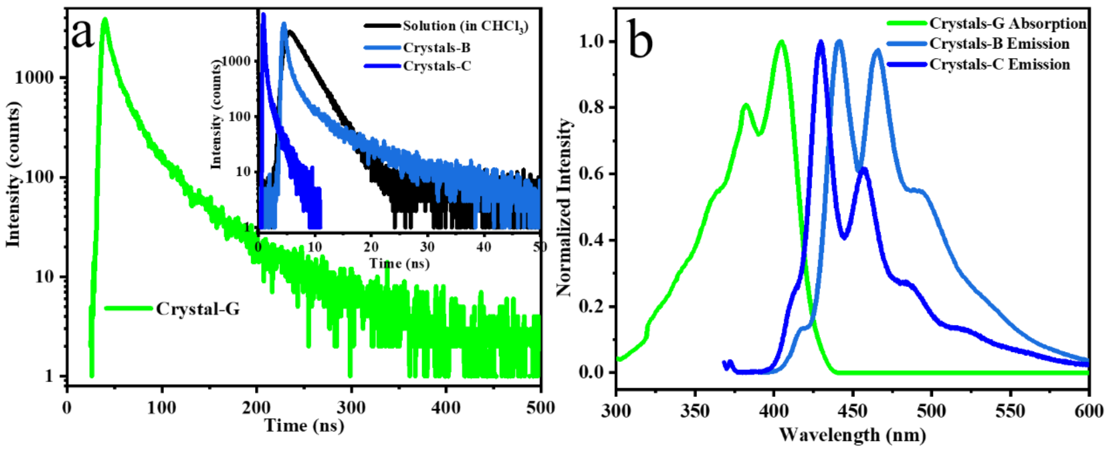


**Figure S6** (a) The photoluminescence decays of solution in $CHCl_3$ and three crystal forms of **5FDPA**. (b) The absorption spectrum of crystals-G and emission spectra of crystals-B and crystals-C.

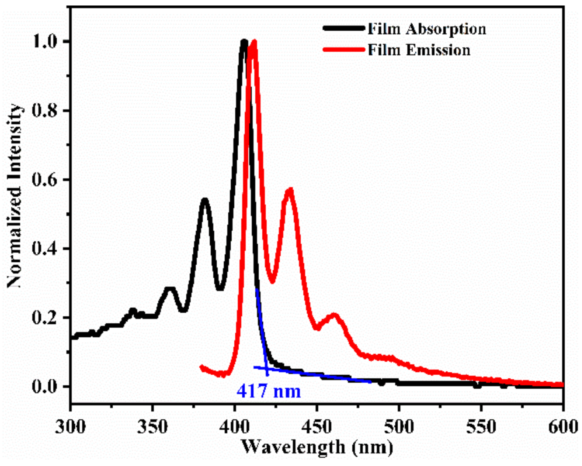


**Figure S7** The absorption and emission spectra of vacuum-deposited thin film of

**5FDPA**.

**Table S1** The photophysical data of **5FDPA** at different states.

| States | $\lambda_{abs}$ (nm) | $\lambda_{em}$ (nm) | Stokes shift (cm$^{-1}$) | $\Phi_F$ (%) | $\tau$ (ns) |
|---|---|---|---|---|---|
| Solution | 334/350/369/391 | 410/435/461/497 | 1185 | 16.2 | 2.55 |
| Thin film | 338/360/382/405 | 411/434/460/495 | 360 | | |
| Crystals-B | 382/405 | 416/440/466/493 | 653 | 52 | 2.01 |
| Crystals-C | 378/403 | 412/429/457/486 | 542 | 43.8 | 0.53 |
| Crystals-G | 382/405 | 508 | 5006 | 62.5 | 21.17 |

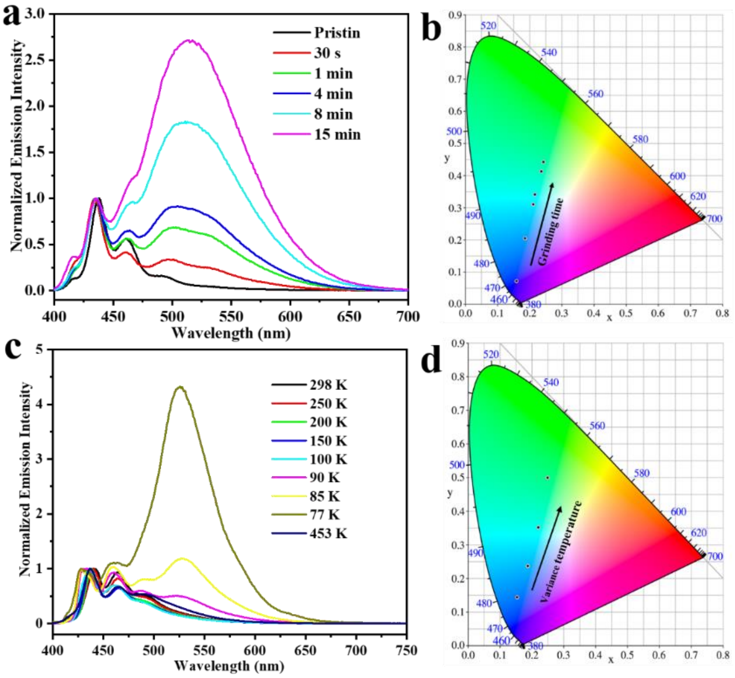


**Figure S8** (a) The fluorescence emission spectra of **5FDPA** after grinding for different times. (b) The change of CIE after grinding for different times. (c) The fluorescence emission spectra of **5FDPA** at different temperatures. (d) The change of CIE at different temperatures.

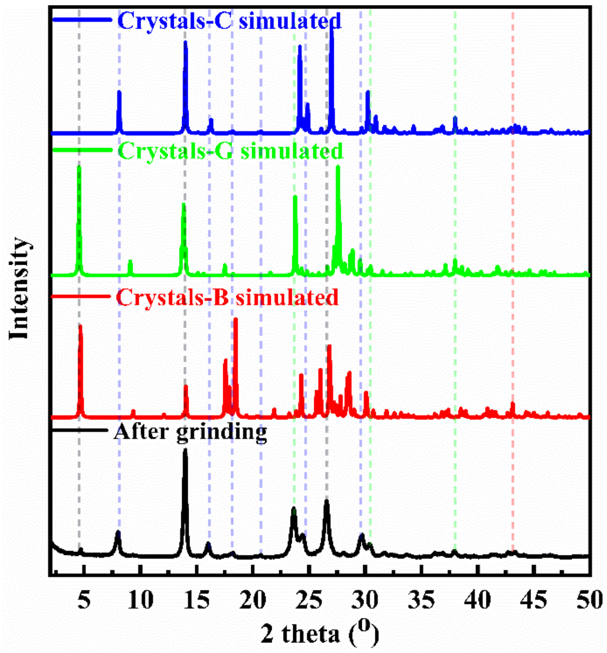


+

**Figure S9** The XRD patterns of **5FDPA** after grinding (black line) and simulated XRD patterns of crystals-B (red line), crystals-G (green line) and crystals-C (blue line).

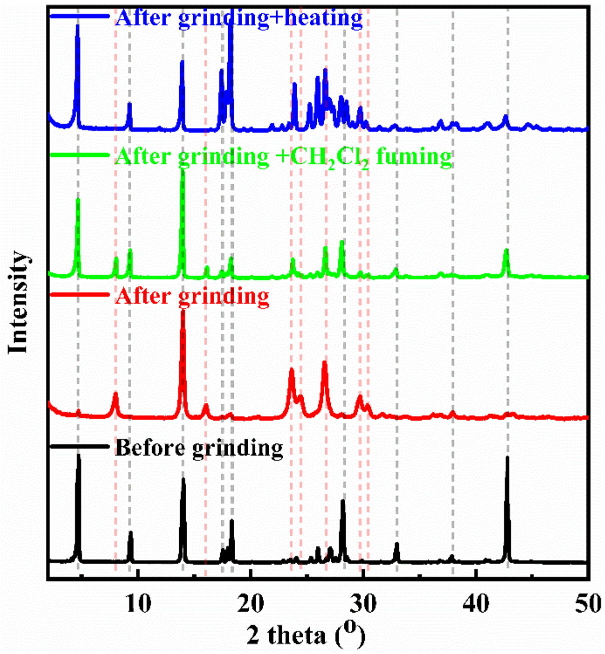


**Figure S10** The XRD patterns of **5FDPA** at different states: before grinding (black line), after grinding (red line), and after grinding, followed by fuming with $CH_2Cl_2$ vapor for 3 min (green line) and heating at 180 $^oC$ for 5 s, respectively.

## 7. Performance of OFETs with vacuum-deposited films of 5FDPA

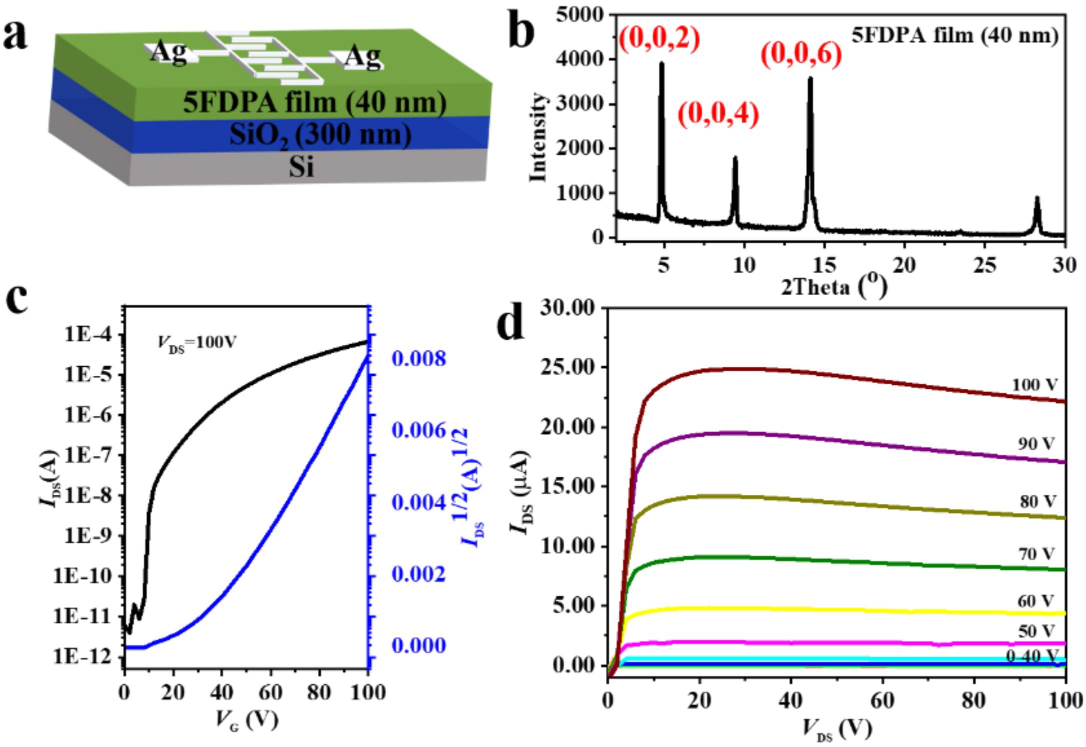


**Figure S11** (a) The device structure of the **5FDPA**-based thin film OFETs. (b) XRD pattern of the thin films (c) Typical transfer curve. (d) Typical output curve.

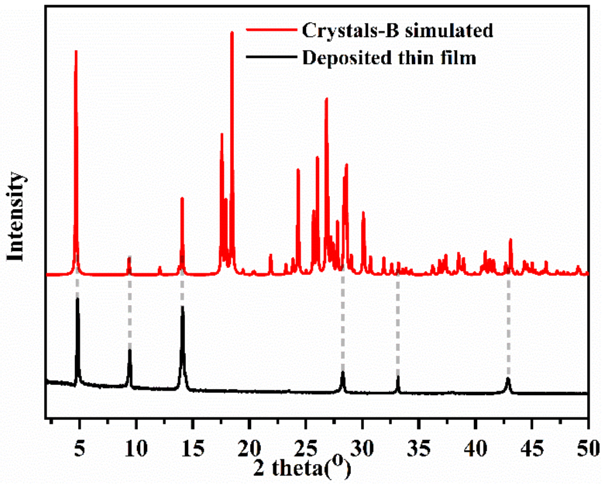


**Figure S12** The XRD pattern of the deposited thin film of **5FDPA** (black) and simulated XRD pattern of crystals-B (red).

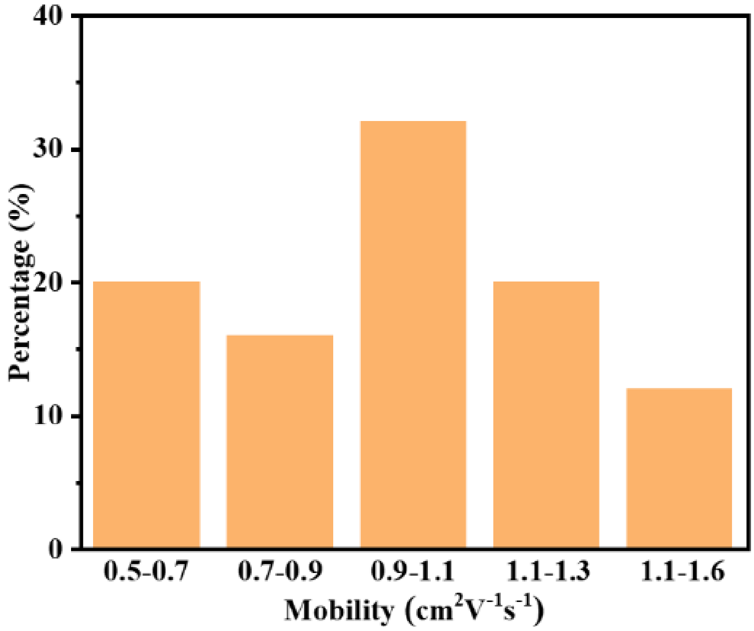


**Figure S13** Electron mobility distribution of 27 devices with the vacuum-deposited films of **5FDPA**.

## 8. Performance of OFETs with crystals of 5FDPP and 5FDPA

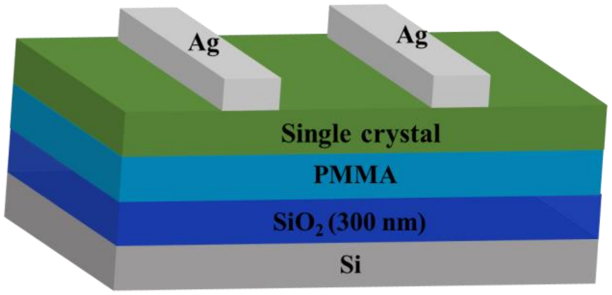


**Figure S14** The device structure of organic single-crystal field-effect transistors based on the crystals of **5FDPP** and **5FDPA**.

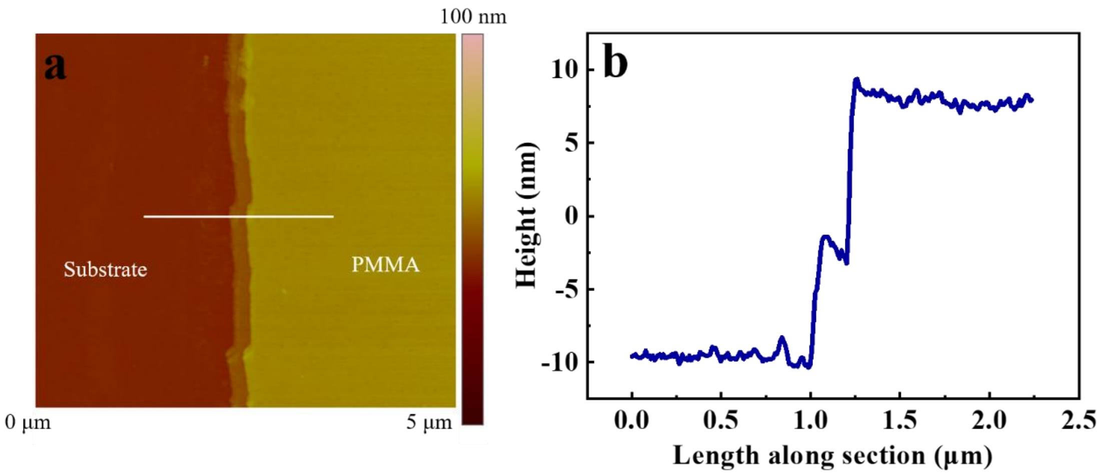


**Figure S15** The Atomic force microscopy (AFM) image (a) and the height file (b) of the PMMA buffer layer.

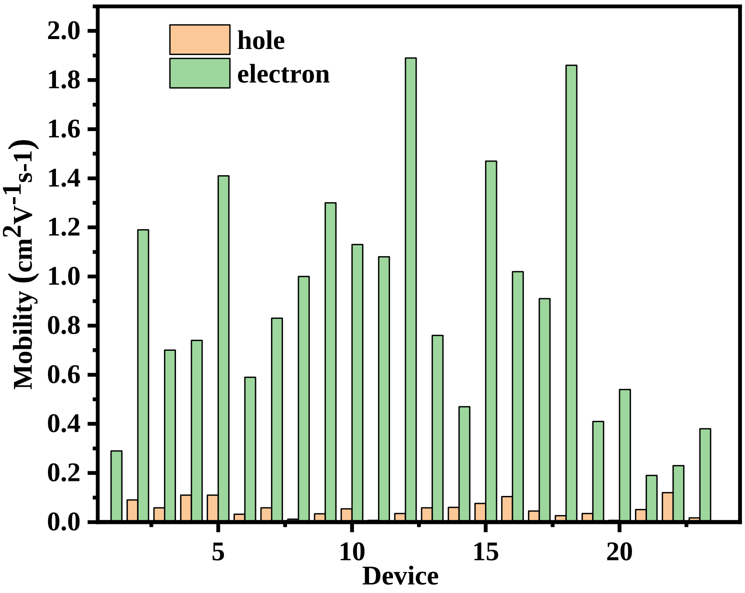

**Figure S16** The hole and electron mobilities of **5FDPP** crystals based on 24 devices.

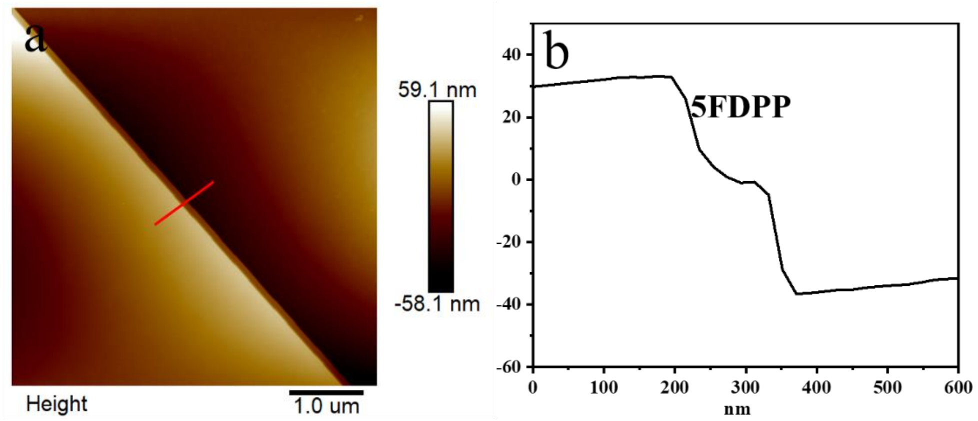


**Figure S17** (a) The atomic force microscopy (AFM) images of crystal of **5FDPP.** (b) The height curve of the crystals measured by AFM. The crystals exhibit very smooth surface with the thickness of ca. 70 nm for **5FDPP**.

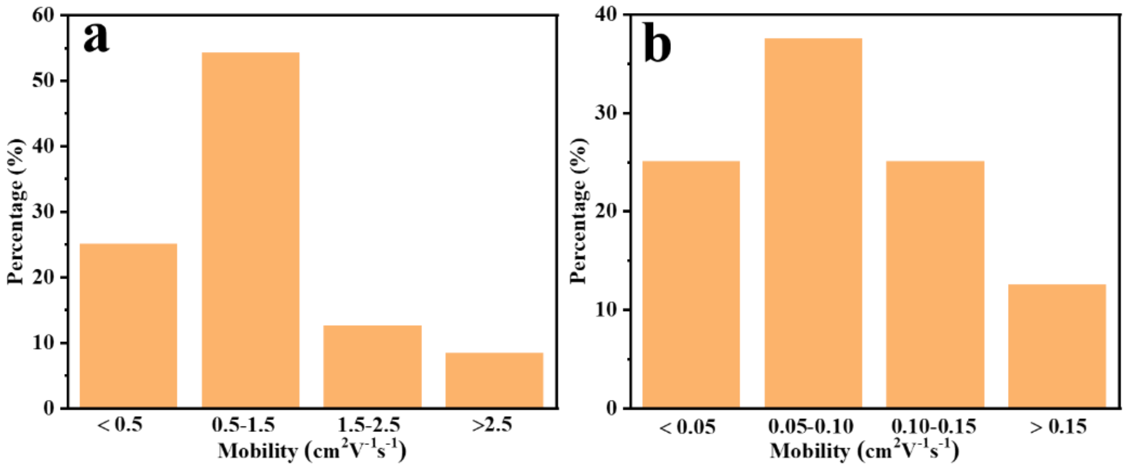


**Figure S18** Electron mobility distribution of single-crystal field-effect transistors: (a) 24 devices with crystals-B; (b) 8 devices with crystals-G.

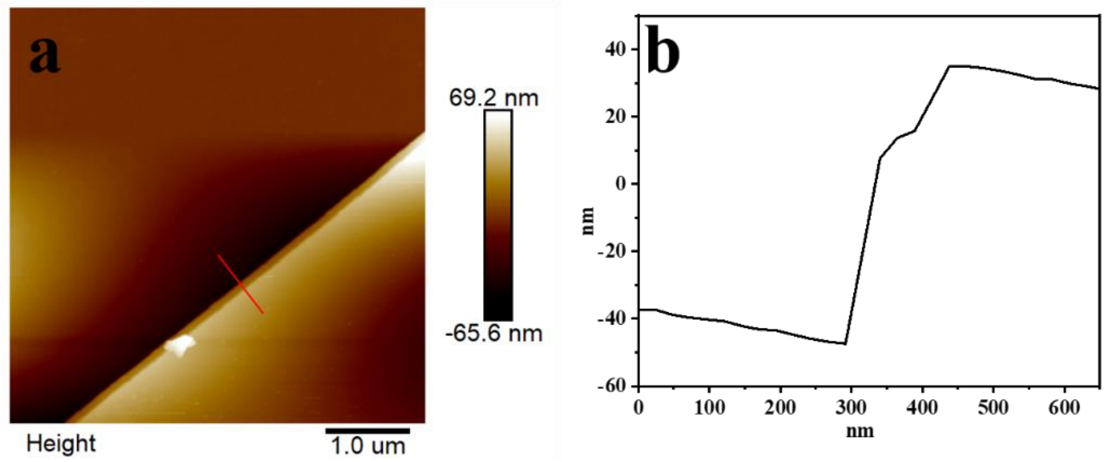


**Figure S19** (a) The AFM image of crystals-B. (b) The height curve of the crystals-B measured by AFM. The crystal exhibits a very smooth surface with a thickness of ca.80 nm.

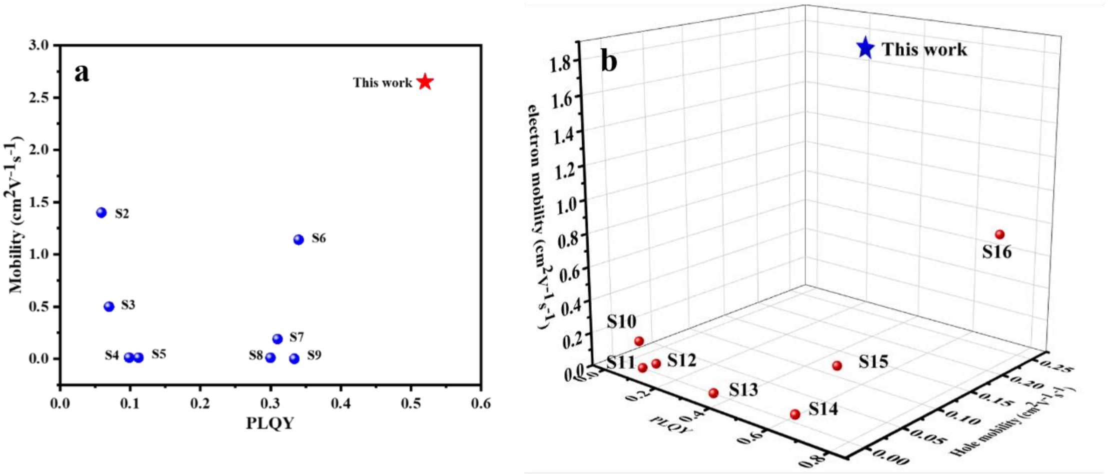


**Figure S20** (a) The electron mobility and PLQY of organic emissive *n*-type semiconductors based on OFET devices reported in the literatures and this work.[S2-9] (b) The mobility and PLQY of organic emissive single-component ambipolar semiconductors based on OFET devices reported in the literatures and this work.[S10-16]


S2. *J. Mater. Chem. C.*, **2018**, *6*, 535-540; S3.*Chem. Mater.*, **2013**, *25*, 668-676.
S4. *Chem. Asian. J.*, **2014**, *9*, 3207-3214; S5. *Macromolecules.*, **2019**, *52*, 8332-8338.
S6. *Adv. Opt. Mater.*, **2019**, *7*, 1901274; S7. *Chem. Mater.*, **2007**, *19*, 1218-1220.
S8. *Adv. Funct. Mater.*, **2018**, *28*, 1705609;
S9. *Chem. Commun.*, **2017**, *53*, 4934-4937.
S10. *J. Phys. Chem. C* **2008**, *112*, 12993–12999;
S11. *J. Mater.Chem. C* **2020**, *8*, 10868.
S12. *Angew. Chem. Int. Ed*. **2021**, *60*, 14902; S13. *J. Appl. Phys*. **2008**, *103*, 064517.
S14. *Adv. Mater*. **2006**, *18*, 2708-2712; S15. *Adv. Mater*. **2008**, *20*, 2217-2224.

S16. *Phys. Chem. Chem. Phys.*, **2015**,*17*, 3421-3425.

Table S2 A summary of device performances for currently reported single-component OLETs based on crystal materials.[S17-26]

| Materials | PLQY(%) | Mobilities ($cm^2V^{-1}s^{-1}$) | | EQE(%) | Electrodes | Ref |
|---|---|---|---|---|---|---|
| | | hole | electron | | | |
| Tetracene | <1 | 2.3 | 0.12 | 0.03 | Au/Ca | S17 |
| Rubrene | <1 | 0.27 | 0.82 | 0.015 | Au/Ca | S17 |
| P5V4 | 40 | 0.1 | 0.1 | 0.1 | Au/Ca | S18 |
| BP3T | 80 | >1 | 0.1 | 1 | Au/Ca | S19 |
| BNF | 55 | 0.1 | 0.04 | 0.27 | Au-MoOx/Ca | S20 |
| NT4N | 13 | 0.007 | 0.55 | 0.2 | Au/Au | S3 |
| DPA | 41 | 1.99 | 0.33 | 1.61 | Au-$MoO_3$/Ca-CsF | S21 |
| dNaAnt | 29 | 0.73 | 1.11 | 1.75 | Au-$MoO_3$/Ca-CsF | S21 |
| PBNA | 82 | 0.18 | 2.71 | 3.63 | Au-$MoO_3$/Ca-CsF | S22 |
| NBTA | 37 | 0.05 | 0.4 | 2.02 | Au-$MoO_3$/Ca-CsF | S23 |
| Hex-4-TFPTA | 28 | | 1.14 | 0.018 | Al-$MoO_3$/Al | S6 |
| TBU-DNA | 75 | 0.48 | 0.16 | 1.8 | Au-$MoO_3$/Ca-CsF | S24 |
| **5FDPP** | **55** | **0.12** | **1.89** | **2.2** | **Ag/Ag** | **This work** |

S17. *Phys. Rev. Lett.* **2008,** *100*, 066601; S18. *Appl. Phys. Lett.* **2009,** *95*, 103307.
S19. *Adv. Mater.* **2012,** *24*, 6141-6146; S20. *Chem. Commun.* **2012,** *48*, 5892-5894.
S21. *Adv. Mater.* **2019**, e1903175; S22. *ACS. Materials. Lett.* **2021,** *3*, 428-432.
S23. *ACS. Appl. Mater. Interfaces.* **2020,** *12*, 43976-43983.
S24. *Angew. Chem. Int. Ed.* **2022,** *61*, e202206825.

## 9. The characterizations of energy levels of 5FDPA and 5FDPP

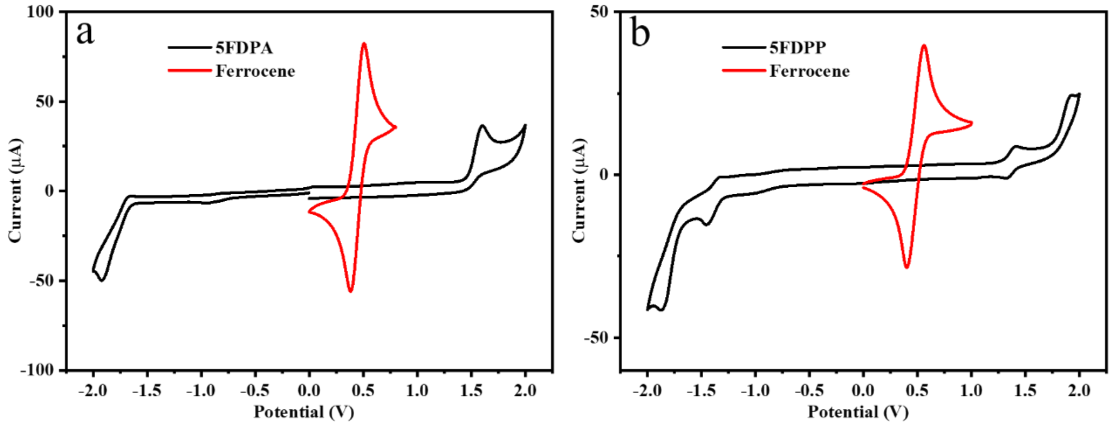


**Figure S21** Cyclic voltammogram of an anhydrous dichloromethane solution of **5FDPA** (a) and **5FDPP** (b) with ferrocene as external standard at a scan rate of 100 $mVs^{-1}$. Glassy carbon electrode was used as working electrode, Pt as counter electrode and Ag/AgCl (saturated KCl) as reference electrode; *n*-$Bu_4NPF_6$ (0.1 M) in $CH_2Cl_2$ as supporting electrolyte.

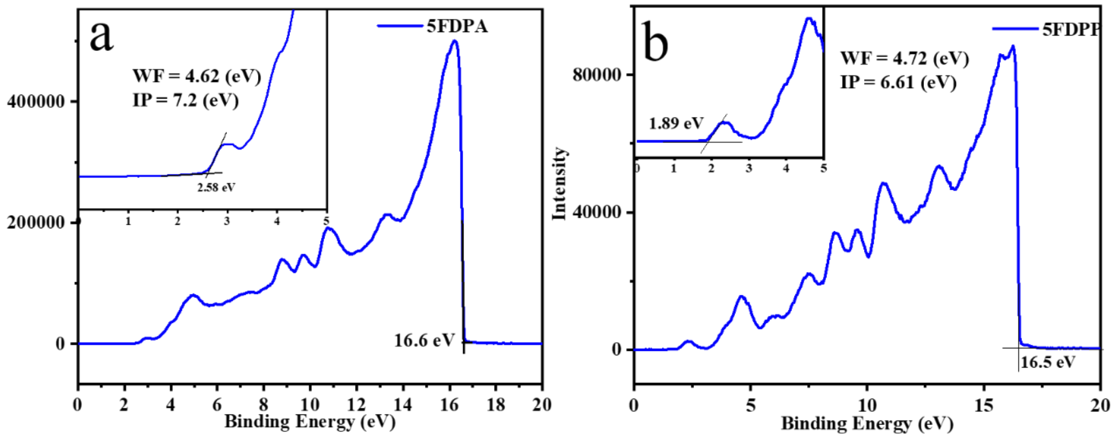


**Figure S22** UPS spectra of **5FDPA** (a) and **5FDPP** (b) based on the deposited thin films**.** **(**WF: work function; IP: ionization potential**)**.

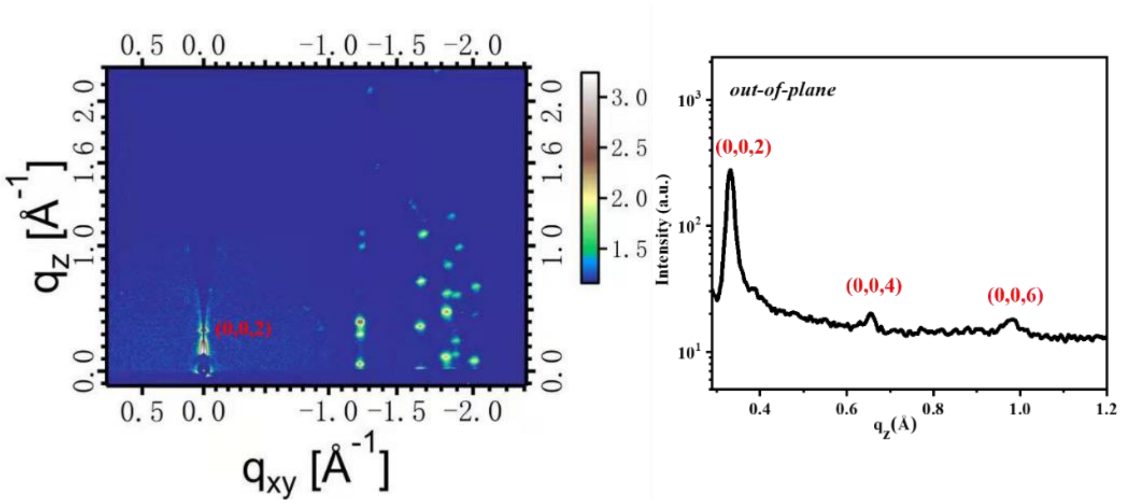


**Figure S23** The GIWAXS (grazing-incidence wide-angle X-ray scattering) image (left) and corresponding out-of-plane cut (right) of vacuum-deposited film of **5FDPA**.

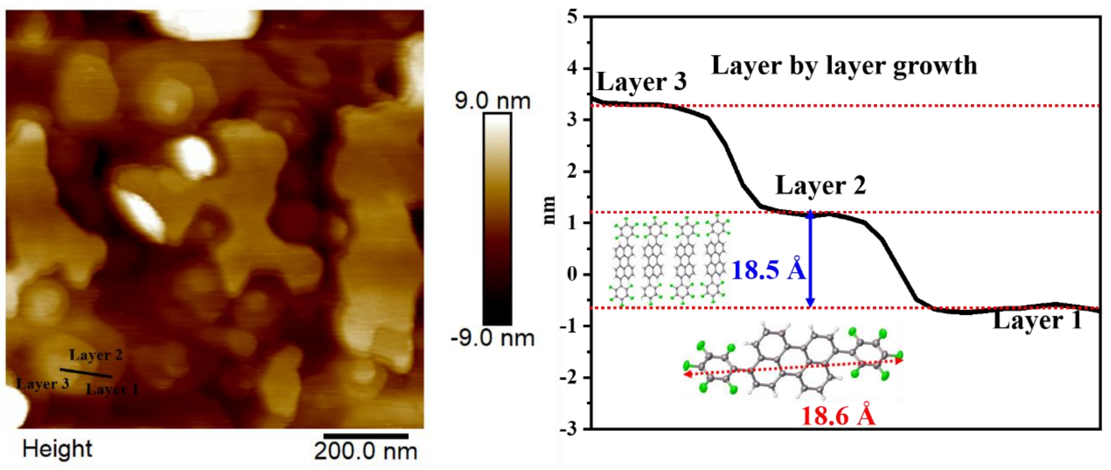


**Figure S24** (a) The atomic force microscopy image of the deposited thin film of **5FDPP**. (b) The step height of layer-by-layer growth of **5FDPP** on the $SiO_2$/Si substrate. The inset shows the molecular length of **5FDPP** in crystal.

## 10. The calculated transfer integrals

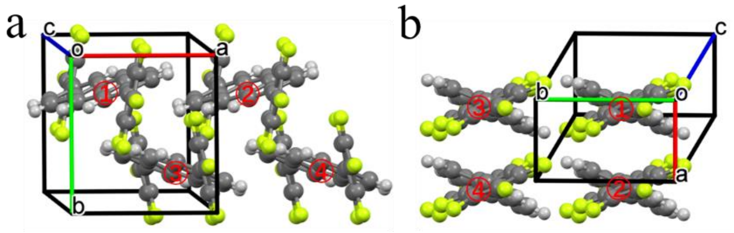


**Crystals-B** **Crystals-G**

| Single-crystal (Dimer) | Crystals-B | | Crystals-G | |
|---|---|---|---|---|
| | 1-2, 3-4 | 1-3, 2-3 | 1-2, 3-4 | 1-3, 2-4 |
| $t_h$ (meV) | 39.5 | 25.5 | 134.8 | 10.7 |
| $t_e$ (meV) | 20.5 | 67.1 | 83.1 | 9.9 |

**Figure S25** The calculated transfer integrals for the nearest neighboring molecules within crystals-B (a) and crystals-G (b).

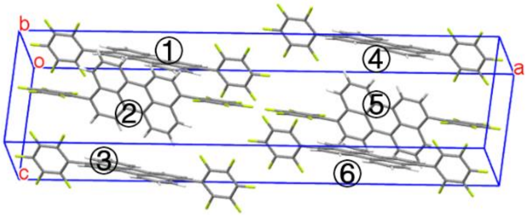


| Dimer | $t_h$ (meV) | $t_e$ (meV) |
|---|---|---|
| 1-2, 2-3 4-5, 5-6 | 2.64 | 23.46 |
| 1-4 | 0.03 | 0.07 |
| 1-5 | 0.47 | 0.80 |

**Figure S26** The calculated transfer integrals for the nearest neighboring molecules within the single crystal of **5FDPP**.

## 11. Performance of OLETs with crystals of 5FDPP and 5FDPA

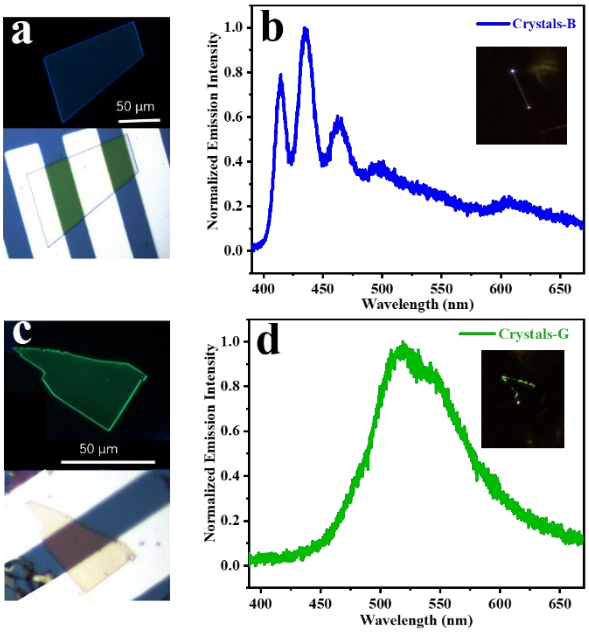


**Figure S27** The photographs of the single-crystal devices with crystals-B (a) and crystals-G (c). The electroluminescence spectra of crystals-B (b) and crystals-G (d). The inset photographs show the corresponding electroluminescence photos.

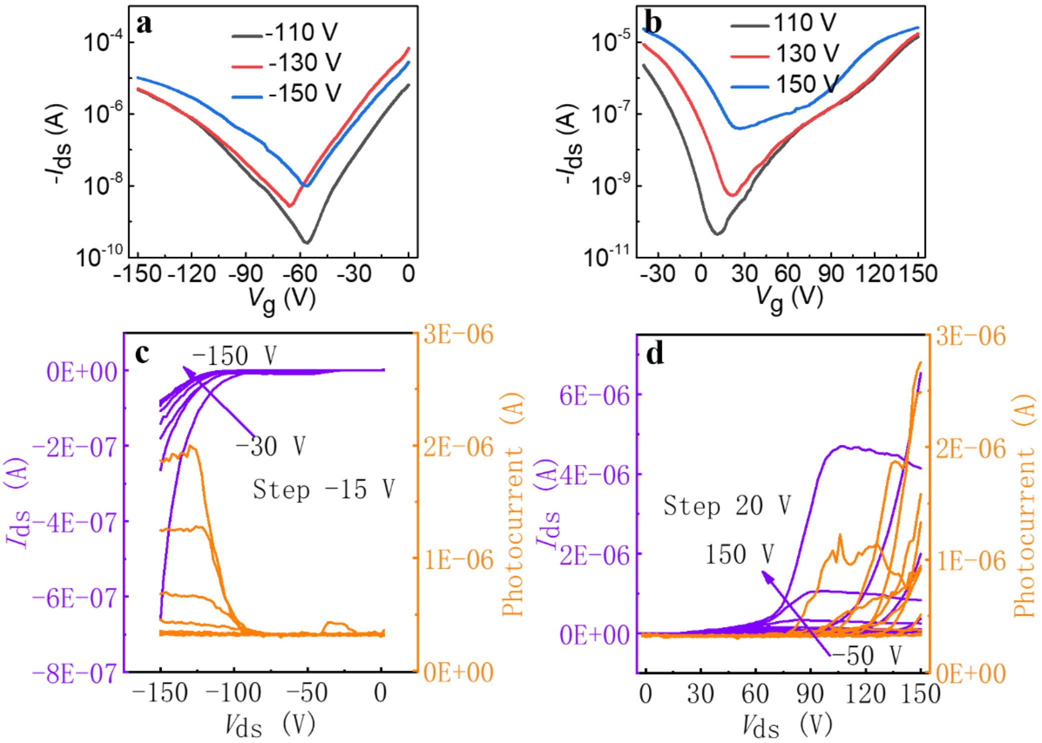


**Figure S28** (a) The transfer curves of **5FDPP**-based OLETs device under negative voltages. (b) The transfer curves of **5FDPP**-based OLETs device under positive voltages. (c) The output curves of **5FDPP**-based OLETs device for *p*-channel. (d) The

output curves of **5FDPP**-based OLETs device for *n*-channel. The data was collected based on the OLETs device without deposition of CuPc on the crystals.

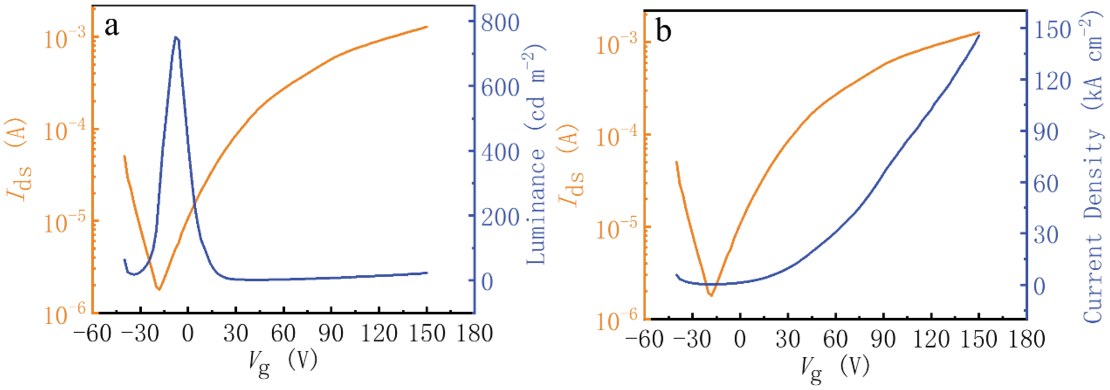


**Figure S29** Electrical and optical transfer characteristics as a function of the gate voltage. (b) Electrical transfer characteristics and current density as a function of the gate voltage. ($V_{ds}$ = 150 V). The maximum current density of a light-emitting transistor is calculated according to the following equation:

$$\text{Current density} = \frac{I_{ds}(maximum)}{W \times L_{OML}}$$

Where $W$ and $L_{OML}$ are the width of the conducting channel and the length of one molecular layer respectively ($W$ = 473μm; $L_{OML}$= 18.6 Å).

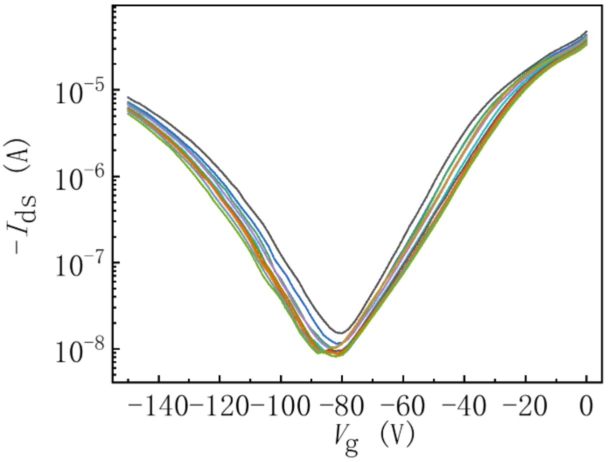


**Figure S30** Transfer curves of the OLETs with 5FDPP crystal after successive measurements for 12 times.

**Table S3** The performances of **5FDPP**-based OLETs devices.

| | Electrode: Ag | | Electrode: CuPc/Ag | |
|---|---|---|---|---|
| Devices | EQE (%) | Mobility ($cm^2V^{-1}s^{-1}$) electron/hole | EQE (%) | Mobility ($cm^2V^{-1}s^{-1}$) electron/hole |
| 1 | 0.62 | 0.092/0.031 | 0.047 | 0.99/0.018 |
| 2 | 1.29 | 0.044/0.012 | 0.018 | 2.5/0.0037 |
| 3 | 0.63 | 0.086/0.0045 | 0.037 | 1.4/0.0032 |
| 4 | 2.2 | 0.06/0.036 | 0.059 | 1/0.033 |
| 5 | 2.02 | 0.12/- | 0.046 | 0.56/0.011 |
| 6 | 0.89 | 0.1/0.033 | 0.025 | 0.22/0.25 |
| 7 | 0.72 | 0.54/0.14 | 0.06 | 1/0.066 |
| 8 | 2 | 0.12/0.011 | 0.1 | 0.1/0.0061 |

## 12. Crystallographic data

**Table S4**. Crystallographic data of **crystals-B**.

| Identification code | **Crystals-B** |
|---|---|
| CCDC deposition NO. | 2164678 |
| Empirical formula | $C_{26}H_8F_{10}$ |
| Formula weight | 510.32 |
| Temperature/K | 170.15 |
| Crystal system | monoclinic |
| Space group | I2/a |
| a/Å | 6.8685(3) |
| b/Å | 7.4556(3) |
| c/Å | 37.8682(17) |
| α/° | 90 |
| β/° | 94.708(4) |
| γ/° | 90 |
| Volume/Å$^3$ | 1932.64(14) |
| Z | 4 |
| $\rho_{calc}$g/cm$^3$ | 1.754 |
| μ/mm$^{-1}$ | 1.516 |
| F(000) | 1016.0 |
| Crystal size/mm$^3$ | 0.2× 0.15 × 0.04 |
| Radiation | CuKα (λ = 1.54184) |
| 2Θ range for data collection/° | 4.682 to 140.532 |
| Index ranges | $-8 \leq h \leq 8$, $-9 \leq k \leq 9$, $-41 \leq l \leq 46$ |
| Reflections collected | 9987 |
| Independent reflections | 1830 [$R_{int}$ = 0.0430, $R_{sigma}$ = 0.0356] |
| Data/restraints/parameters | 1830/0/163 |
| Goodness-of-fit on $F^2$ | 1.088 |
| Final R indexes [I>=2σ (I)] | $R_1$ = 0.0528, $wR_2$ = 0.1489 |
| Final R indexes [all data] | $R_1$ = 0.0674, $wR_2$ = 0.1590 |
| Largest diff. peak/hole / e Å$^{-3}$ | 0.49/-0.31 |

**Table S5**. Crystallographic data of **crystals-C**.

| Identification code | **Crystals-C** |
|---|---|
| CCDC deposition NO. | 2164674 |
| Empirical formula | $C_{13}H_4F_5$ |
| Formula weight | 255.17 |
| Temperature/K | 169.99(12) |
| Crystal system | monoclinic |
| Space group | C2/c |
| a/Å | 22.6177(4) |
| b/Å | 6.59380(10) |
| c/Å | 13.3302(2) |
| α/° | 90 |
| β/° | 105.983(2) |
| γ/° | 90 |
| Volume/Å$^3$ | 1911.17(6) |
| Z | 8 |
| $\rho_{calc}$g/cm$^3$ | 1.7735 |
| μ/mm$^{-1}$ | 1.532 |
| F(000) | 1020.9 |
| Crystal size/mm$^3$ | 0.21× 0.12 × 0.05 |
| Radiation | CuKα (λ = 1.54184) |
| 2Θ range for data collection/° | 8.14 to 135.88 |
| Index ranges | $-27 \le h \le 28$, $-7 \le k \le 5$, $-16 \le l \le 16$ |
| Reflections collected | 5851 |
| Independent reflections | 1726 [$R_{int}$ = 0.0180, $R_{sigma}$ = 0.0173] |
| Data/restraints/parameters | 1726/0/163 |
| Goodness-of-fit on F$^2$ | 1.050 |
| Final R indexes [I>=2σ (I)] | $R_1$ = 0.0283, $wR_2$ = 0.0777 |
| Final R indexes [all data] | $R_1$ = 0.0319, $wR_2$ = 0.0787 |
| Largest diff. peak/hole / e Å$^{-3}$ | 0.35/-0.38 |

**Table S6**. Crystallographic data of **crystals-G**.

| Identification code | **Crystals-G** |
|---|---|
| CCDC deposition NO. | 2164676 |
| Empirical formula | $C_{13}H_4F_5$ |
| Formula weight | 255.16 (10) |
| Temperature/K | 169.99(10) |
| Crystal system | triclinic |
| Space group | P-1 |
| a/Å | 3.7710(2) |
| b/Å | 6.4409(3) |
| c/Å | 19.5947(9) |
| α/° | 97.053(4) |
| β/° | 93.355(4) |
| γ/° | 90.652(4) |
| Volume/$Å^3$ | 471.43(4) |
| Z | 2 |
| $\rho_{calc}g/cm^3$ | 1.789 |
| $\mu/mm^{-1}$ | 1.553 |
| F(000) | 254.0 |
| Crystal size/$mm^3$ | 0.2× 0.03 × 0.02 |
| Radiation | CuKα (λ = 1.54184) |
| 2Θ range for data collection/° | 4.552 to 149.794 |
| Index ranges | $-4 \le h \le 4$, $-8 \le k \le 8$, $-21 \le l \le 24$ |
| Reflections collected | 5254 |
| Independent reflections | 1840 [$R_{int}$ = 0.0247, $R_{sigma}$ = 0.0227] |
| Data/restraints/parameters | 1840/0/163 |
| Goodness-of-fit on $F^2$ | 1.083 |
| Final R indexes [I>=2σ (I)] | $R_1$ = 0.0492, $wR_2$ = 0.1465 |
| Final R indexes [all data] | $R_1$ = 0.0542, $wR_2$ = 0.1521 |
| Largest diff. peak/hole / e $Å^{-3}$ | 0.49/-0.31 |

**Table S7**. Crystallographic data of **crystals-B′**.

| Identification code | **Crystals-B′** |
|---|---|
| CCDC deposition NO. | 2164677 |
| Empirical formula | $C_{26}H_8F_{10}$ |
| Formula weight | 510.32 |
| Temperature/K | 109.99(11) |
| Crystal system | monoclinic |
| Space group | C2/c |
| a/Å | 37.783(2) |
| b/Å | 7.4314(4) |
| c/Å | 6.8606(3) |
| α/° | 90 |
| β/° | 95.205(5) |
| γ/° | 90 |
| Volume/Å$^3$ | 1918.38(17) |
| Z | 4 |
| $\rho_{calc}$g/cm$^3$ | 1.767 |
| μ/mm$^{-1}$ | 1.527 |
| F(000) | 1016.0 |
| Crystal size/mm$^3$ | 0.18× 0.16 × 0.05 |
| Radiation | CuKα (λ = 1.54184) |
| 2Θ range for data collection/° | 4.696 to 149.452 |
| Index ranges | -46 ≤ h ≤ 46, -9 ≤ k ≤ 9, -7 ≤ l ≤ 8 |
| Reflections collected | 10466 |
| Independent reflections | 1904 [$R_{int}$ = 0.0540, $R_{sigma}$ = 0.0187] |
| Data/restraints/parameters | 1904/0/163 |
| Goodness-of-fit on F$^2$ | 1.100 |
| Final R indexes [I>=2σ (I)] | $R_1$ = 0.0799, w$R_2$ = 0.2256 |
| Final R indexes [all data] | $R_1$ = 0.0818, w$R_2$ = 0.2271 |

| | |
|---|---|
| Largest diff. peak/hole / e Å$^{-3}$ | 0.46/-0.32 |

**Table S8**. Crystallographic data of **5FDPP**.

| Identification code | **5FDPP** |
|---|---|
| CCDC deposition NO. | 2178552 |
| Empirical formula | $C_{32}H_{10}F10$ |
| Formula weight | 584.40 |
| Temperature/K | 170.15 |
| Crystal system | monoclinic |
| Space group | C2/c |
| a/Å | 37.4999(14) |
| b/Å | 6.4177(2) |
| c/Å | 9.5179(4) |
| α/° | 90 |
| β/° | 91.060(3) |
| γ/° | 90 |
| Volume/Å$^3$ | 2290.22(15) |
| Z | 4 |
| $\rho_{calc}$g/cm$^3$ | 1.695 |
| μ/mm$^{-1}$ | 1.373 |
| F(000) | 1168.0 |
| Crystal size/mm$^3$ | 0.25× 0.13 × 0.05 |
| Radiation | CuKα (λ = 1.54184) |
| 2Θ range for data collection/° | 4.714 to 154.206 |
| Index ranges | $-47 \le h \le 44$, $-8 \le k \le 4$, $-11 \le l \le 10$ |
| Reflections collected | 7222 |
| Independent reflections | 2308 [$R_{int}$ = 0.00355, $R_{sigma}$ = 0.0339] |
| Data/restraints/parameters | 2308/0/190 |
| Goodness-of-fit on F$^2$ | 1.061 |
| Final R indexes [I>=2σ (I)] | $R_1$ = 0.0447, $wR_2$ = 0.1247 |
| Final R indexes [all data] | $R_1$ = 0.0507, $wR_2$ = 0.1301 |
| Largest diff. peak/hole / e Å$^{-3}$ | 0.34/-0.23 |

## 13. $^{1}$H NMR, $^{13}$C NMR and $^{19}$F NMR spectra

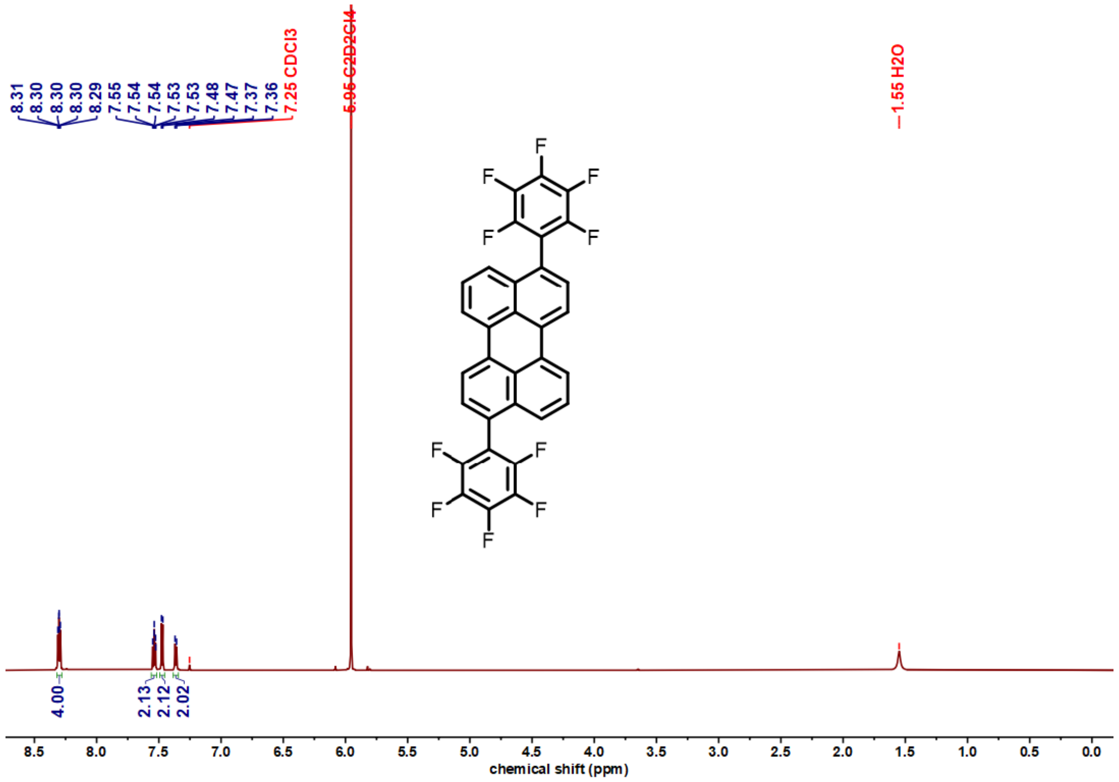


**Figure S31** $^{1}$H NMR spectrum of compound **5FDPP** (700 MHz, $C_2D_2Cl_4$, 373 K).

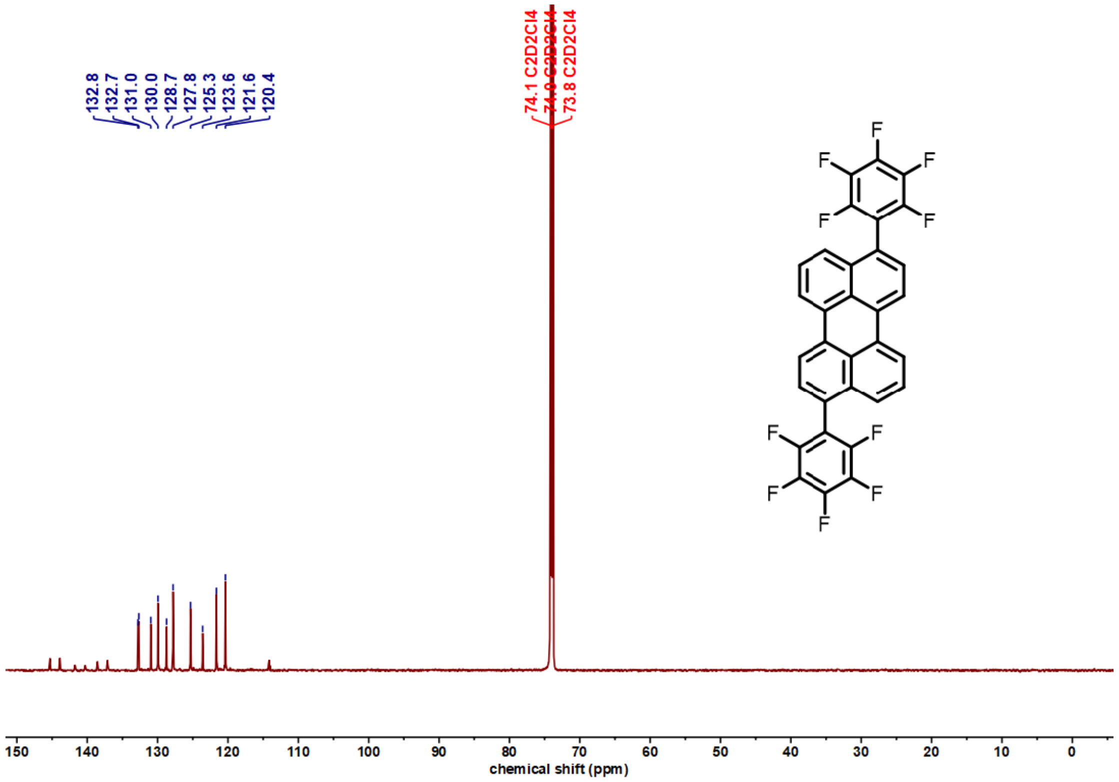


**Figure S32** $^{13}$C NMR spectrum of compound **5FDPP** (176 MHz, $C_2D_2Cl_4$, 373 K, decoupling of $^{1}$H).

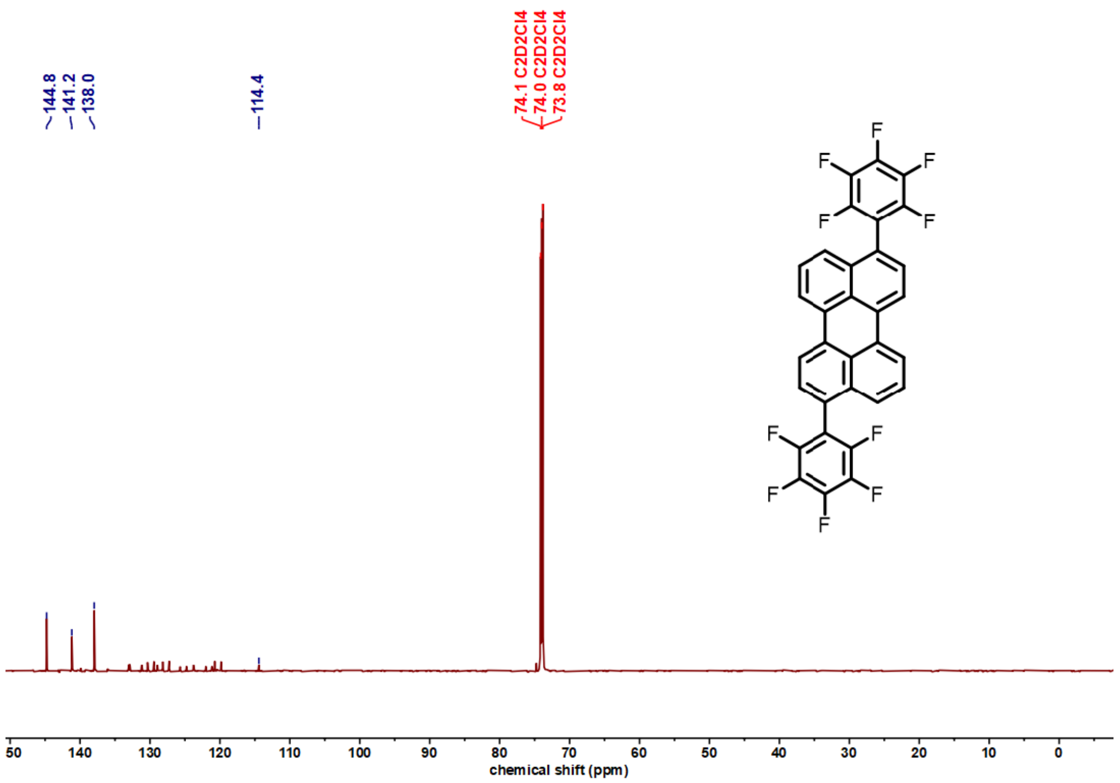


**Figure S33** $^{13}$C NMR spectrum of compound **5FDPP** (176 MHz, $C_2D_2Cl_4$, 373 K, decoupling of $^{19}$F).

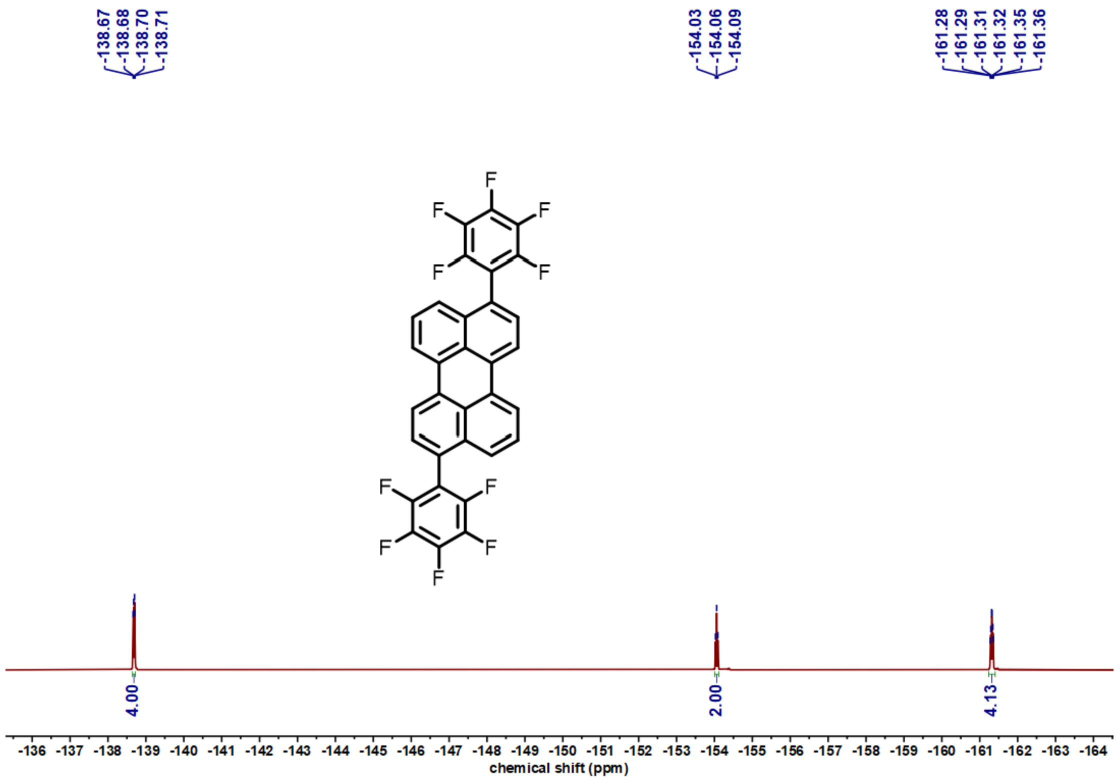


**Figure S34** $^{19}$F NMR spectrum of compound **5FDPP** (659 MHz, $C_2D_2Cl_4$, 373 K).

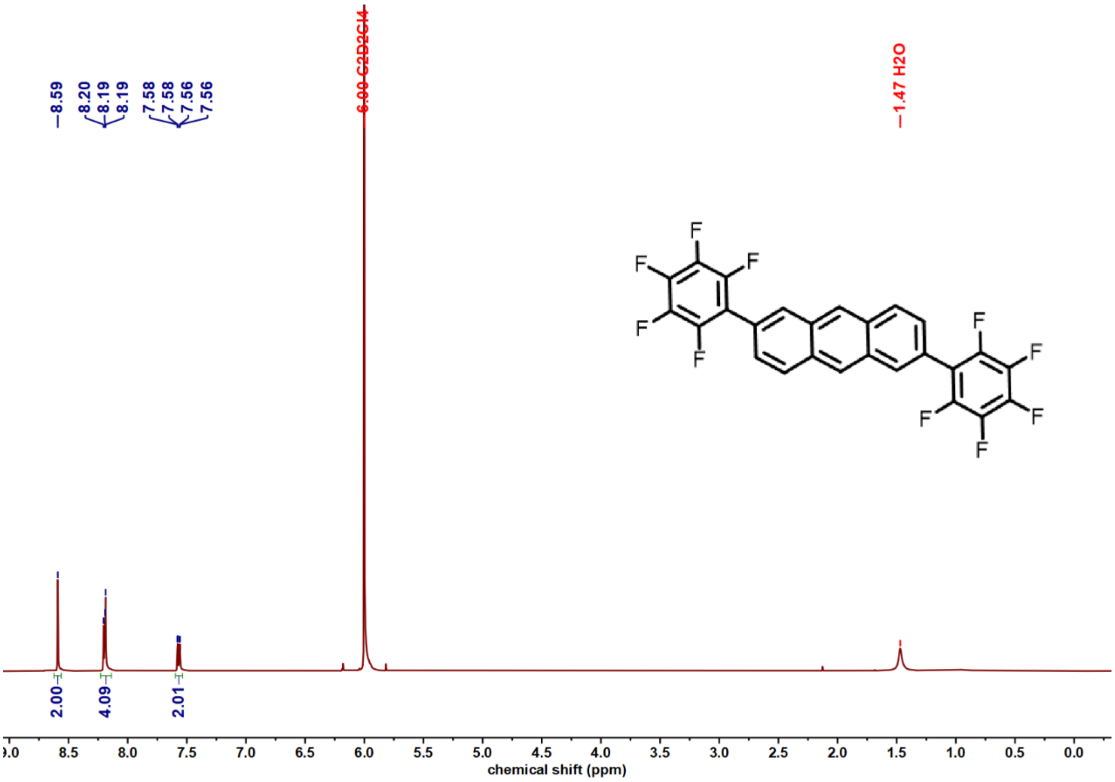


**Figure S35** $^{1}$H NMR spectrum of compound **5FDPA** (500 MHz, $C_2D_2Cl_4$, 373 K).

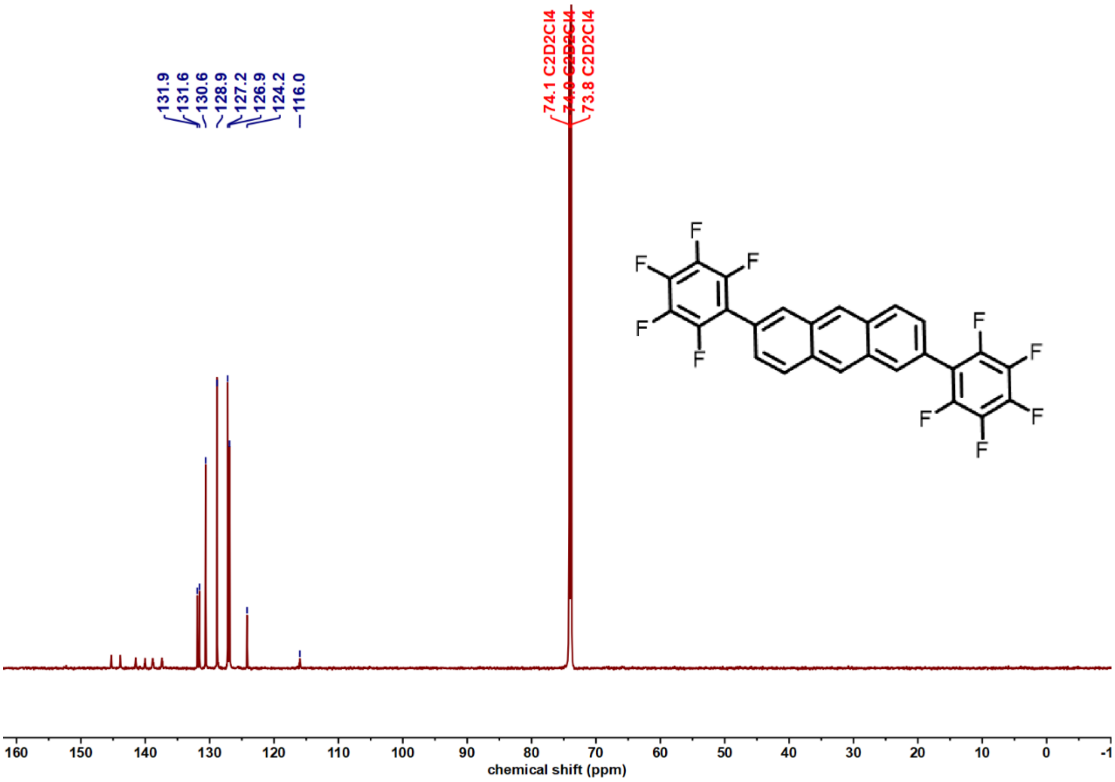


**Figure S36** $^{13}$C NMR spectrum of compound **5FDPA** (176 MHz, $C_2D_2Cl_4$, 373 K, decoupling of $^{1}$H).

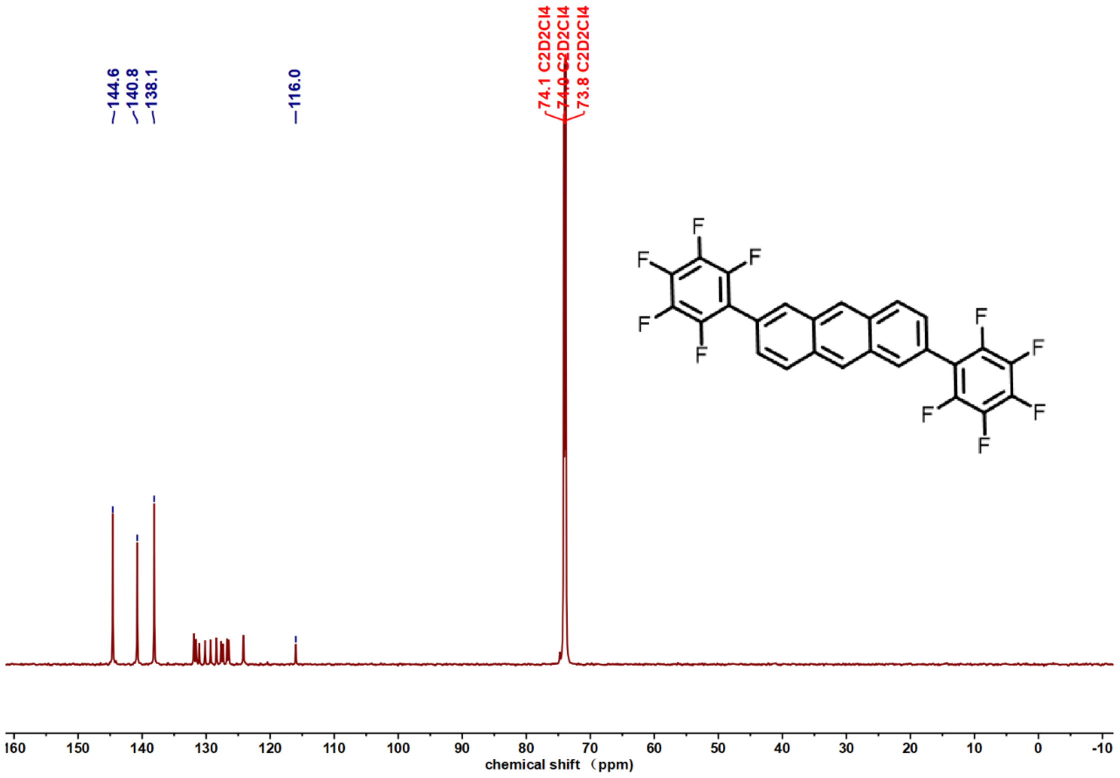


**Figure S37** $^{13}$C NMR spectrum of compound **5FDPA** (176 MHz, $C_2D_2Cl_4$, 373 K, decoupling of $^{19}$F).

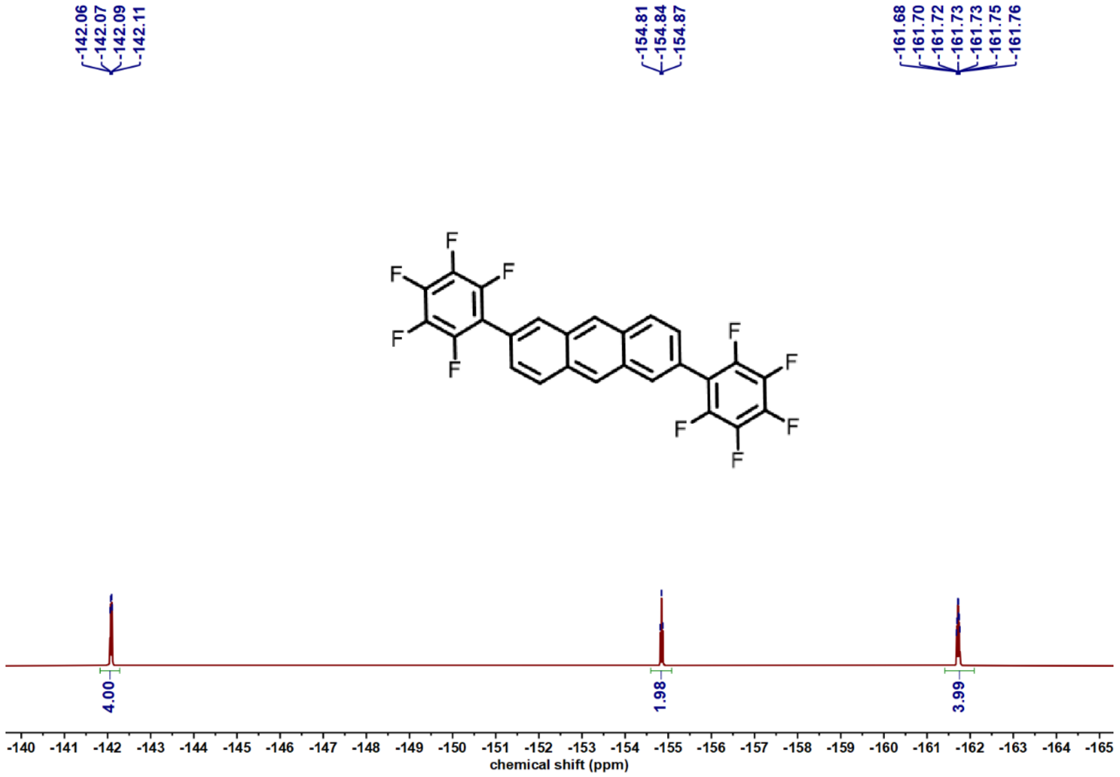


**Figure S38** $^{19}$F NMR spectrum of compound **5FDPA** (659 MHz, $C_2D_2Cl_4$, 373 K).

## 14. References


S1. Ye, X.; Liu, Y.; Han, Q.; Ge, C.; Cui, S.; Zhang, L.; Zheng, X.; Liu, G.; Liu, J.; Liu, D.; Tao, X., Microspacing In-Air Sublimation Growth of Organic Crystals. *Chem. Mater.* **2018**, *30*, 412-420.

S2. Ma, L.; Qin, D.; Liu, Y.; Zhan, X., n-Type organic light-emitting transistors with high mobility and improved air stability. *J. Mater. Chem. C.* **2018**, *6*, 535-540.

S3. Melucci, M.; Favaretto, L.; Zambianchi, M.; Durso, M.; Gazzano, M.; Zanelli, A.; Monari, M.; Lobello, M. G.; De Angelis, F.; Biondo, V.; Generali, G.; Troisi, S.; Koopman, W.; Toffanin, S.; Capelli, R.; Muccini, M., Molecular Tailoring of New Thieno(bis)imide-Based Semiconductors for Single Layer Ambipolar Light Emitting Transistors. *Chem. Mater.* **2013**, *25*, 668-676.

S4. Li, Y.; Zhang, G.; Zhang, W.; Wang, J.; Chen, X.; Liu, Z.; Yan, Y.; Zhao, Y.; Zhang, D., Arylacetylene-substituted naphthalene diimides with dual functions: optical waveguides and n-type semiconductors. *Chem. Asian. J.* **2014**, *9*, 3207-14.

S5. Chen, Y.; Liang, X.; Yang, H.; Wang, Q.; Zhou, X.; Guo, D.; Li, S.; Zhou, C.; Dong, L.; Liu, Z.; Cai, Z.; Chen, W.; Tan, L., Strong Near-Infrared Solid Emission and Enhanced N-Type Mobility for Poly(naphthalene Diimide) Vinylene by a Random Polymerization Strategy. *Macromolecules.*, **2019**, *52*, 8332-8338.

S6. Oh, S.; Kim, J. H.; Park, S. K.; Ryoo, C. H.; Park, S. Y., Fabrication of Pixelated Organic Light-Emitting Transistor (OLET) with a Pure Red-Emitting Organic Semiconductor. *Adv. Opt. Mater.* **2019**, *7*, 1901274.

S7. Kono, T.; Kumaki, D.; Nishida, J.-i.; Sakanoue, T.; Kakita, M.; Tada, H.; Tokito, S.; Yamashita, Y., High-performance and light-emitting n-type organic field-effect transistors based on dithienylbenzothiadiazole and related heterocycles. *Chem. Mater.* **2007**, *19*, 1218-1220.

S8. Zhao, Z.; Gao, S.; Zheng, X.; Zhang, P.; Wu, W.; Kwok, R. T. K.; Xiong, Y.; Leung, N. L. C.; Chen, Y.; Gao, X.; Lam, J. W. Y.; Tang, B. Z., Rational Design of Perylenediimide-Substituted Triphenylethylene to Electron Transporting Aggregation-Induced Emission Luminogens (AIEgens) with High Mobility and Near-Infrared Emission. *Adv. Funct. Mater.* **2018**, *28*, 1705609.

S9. Liang, X.; Tan, L.; Liu, Z.; Ma, Y.; Zhang, G.; Wang, L.; Li, S.; Dong, L.; Li, J.; Chen, W., Poly(naphthalene diimide) vinylene: solid state red emission and semiconducting properties for transistors. *Chem. Commun.* **2017**, *53*, 4934-4937.

S10. Capelli, R.; Dinelli, F.; Toffanin, S.; Todescato, F.; Murgia, M.; Muccini, M.; Facchetti, A.; Marks, T. J., Investigation of the optoelectronic properties of organic light-emitting transistors based on an intrinsically ambipolar material. *J. Phys. Chem. C.* **2008,** *112*, 12993-12999.

S11. Zhang, Y.; Ye, J.; Liu, Z.; Liu, Q.; Guo, X.; Dang, Y.; Zhang, J.; Wei, Z.; Wang, Z.; Wang, Z.; Dong, H.; Hu, W. Red-emissive poly(phenylene vinylene)-derivated semiconductors with well-balanced ambipolar electrical transporting properties. *J. Mater. Chem. C.* **2020,** *8*, 10868-10879.

S12. Guo, X.; Zhang, Y.; Hu, Y.; Yang, J.; Li, Y.; Ni, Z.; Dong, H.; Hu, W. Molecular Weight Engineering in High-Performance Ambipolar Emissive Mesopolymers. *Angew.*

*Chem. Int. Ed.* **2021,** *60*, 14902-14908.
S13. Zaumseil, J.; McNeill, C. R.; Bird, M.; Smith, D. L.; Paul Ruden, P.; Roberts, M.; McKiernan, M. J.; Friend, R. H.; Sirringhaus, H. Quantum efficiency of ambipolar light-emitting polymer field-effect transistors. *J. Appl. Phys.* **2008,** *103*, 064517.
S14. Zaumseil, J.; Donley, C. L.; Kim, J. S.; Friend, R. H.; Sirringhaus, H. Efficient Top-Gate, Ambipolar, Light-Emitting Field-Effect Transistors Based on a Green-Light-Emitting Polyfluorene. *Adv. Mater.* **2006,** *18*, 2708-2712.
S15. Bürgi, L.; Turbiez, M.; Pfeiffer, R.; Bienewald, F.; Kirner, H.-J.; Winnewisser, C. High-Mobility Ambipolar Near-Infrared Light-Emitting Polymer Field-Effect Transistors. *Adv. Mater.* **2008,** *20*, 2217-2224.
S16. Deng, J.; Tang, J.; Xu, Y.; Liu, L.; Wang, Y.; Xie, Z.; Ma, Y. Cyano-substituted oligo(p-phenylene vinylene) single-crystal with balanced hole and electron injection and transport for ambipolar field-effect transistors. *Phys. Chem. Chem. Phys.* **2015,** *17*, 3421-3425.
S17. Takenobu, T.; Bisri, S. Z.; Takahashi, T.; Yahiro, M.; Adachi, C.; Iwasa, Y., High current density in light-emitting transistors of organic single crystals. *Phys. Rev. Lett.* **2008,** *100*, 066601.
S18. Nakanotani, H.; Saito, M.; Nakamura, H.; Adachi, C., Tuning of threshold voltage by interfacial carrier doping in organic single crystal ambipolar light-emitting transistors and their bright electroluminescence. *Appl. Phys. Lett.* **2009,** *95*, 103307.
S19. Sawabe, K.; Imakawa, M.; Nakano, M.; Yamao, T.; Hotta, S.; Iwasa, Y.; Takenobu, T., Current-confinement structure and extremely high current density in organic light-emitting transistors. *Adv. Mater.* **2012,** *24*, 6141-6146.
S20. Niimi, K.; Mori, H.; Miyazaki, E.; Osaka, I.; Kakizoe, H.; Takimiya, K.; Adachi, C., [2,2']Bi[naphtho[2,3-b]furanyl]: a versatile organic semiconductor with a furan-furan junction. *Chem. Commun.* **2012,** *48*, 5892-5894.
S21. Qin, Z.; Gao, H.; Liu, J.; Zhou, K.; Li, J.; Dang, Y.; Huang, L.; Deng, H.; Zhang, X.; Dong, H.; Hu, W., High-Efficiency Single-Component Organic Light-Emitting Transistors. *Adv. Mater.* **2019**, e1903175.
S22. Liu, L.; Cai, C.; Zhang, Z.; Zhang, S.; Deng, J.; Yang, B.; Gu, C.; Ma, Y., Lamellar Organic Light-Emitting Crystals Exhibiting Spectral Gain and 3.6% External Quantum Efficiency in Transistors. *ACS Materials Lett.* **2021,** *3*, 428-432.
S23. Wan, Y.; Deng, J.; Wu, W.; Zhou, J.; Niu, Q.; Li, H.; Yu, H.; Gu, C.; Ma, Y., Efficient Organic Light-Emitting Transistors Based on High-Quality Ambipolar Single Crystals. *ACS Appl Mater Interfaces.* **2020,** *12*, 43976-43983.
S24. Li, J.; Qin, Z.; Sun, Y.; Zhen, Y.; Liu, J.; Zou, Y.; Li, C.; Lu, X.; Jiang, L.; Zhang, X.; Ji, D.; Li, L.; Dong, H.; Hu, W., Regulating Crystal Packing by Terminal tert-Butylation for Enhanced Solid-State Emission and Efficacious Charge Transport in an Anthracene-Based Molecular Crystal. *Angew. Chem. Int. Ed.* **2022,** *61*, e202206825.
